# Ethnobotanical study on wild edible plants used by three trans-boundary ethnic groups in Jiangcheng County, Pu’er, Southwest China

**DOI:** 10.1186/s13002-020-00420-1

**Published:** 2020-10-27

**Authors:** Yilin Cao, Ren Li, Shishun Zhou, Liang Song, Ruichang Quan, Huabin Hu

**Affiliations:** 1 Agriculture Service Center, Zhengdong Township, Pu’er City, 665903 Yunnan China; 2 Southeast Asia Biodiversity Research Institute, Chinese Academy of Sciences & Center for Integrative Conservation, Xishuangbanna Tropical Botanical Garden, Chinese Academy of Sciences, Mengla, 666303 Yunnan China; 3 University of Chinese Academy of Sciences, Beijing, 100049 China; 4 CAS Key Laboratory of Tropical Forest Ecology,Xishuangbanna Tropical Botanical Garden, Chinese Academy of Sciences, Menglun, 666303 Yunnan China; 5 CAS Key Laboratory of Tropical Plant Resources and Sustainable Use, Xishuangbanna Tropical Botanical Garden, Chinese Academy of Sciences, Mengla, 666303 Yunnan China

**Keywords:** Wild edible plants, Trans-boundary ethnic groups, Traditional knowledge, Conservation and sustainable use, Jiangcheng County

## Abstract

**Background:**

Dai, Hani, and Yao people, in the trans-boundary region between China, Laos, and Vietnam, have gathered plentiful traditional knowledge about wild edible plants during their long history of understanding and using natural resources. The ecologically rich environment and the multi-ethnic integration provide a valuable foundation and driving force for high biodiversity and cultural diversity in this region. However, little study has uncovered this unique and attractive culture to the world.

**Methods:**

We conducted ethnobotanical survey in 20 villages of Jiangcheng County from 2016 to 2020. Altogether 109 local Dai, Hani, and Yao people were interviewed, and their traditional knowledge about wild edible plants was recorded. Voucher specimens were identified by the authors and deposited in the herbarium of Xishuangbanna Tropical Botanical Garden, Chinese Academy of Sciences (HITBC). The use value was used as a quantitative index to evaluate the consumption frequency and relative importance of the wild edible plants. The Jaccard index was calculated to assess the usage similarity of different areas. The relationship of age and recognized wild edible plants by different ethnic people was performed by R.

**Results:**

A total of 211 wild edible plants, belonging to 71 families and 151 genera, were recorded. These plants were consumed as wild edible vegetables, seasonal fruits, salads, spices, sour condiments, tonic soups, tea substitutes, liquor brewing, or dyeing materials. The use value (UV), current cultivation, market availability, and the quantitative traditional knowledge inheritance situation of these wild edible plants among different generations, were analyzed. Based on the data from the threatened species list of China’s higher plants and the IUCN Red List, the food plant list for Asia Elephant, the Subject Database of China Plant, and the calculated UV score, the top 30 most important wild edible plants were selected for further cultivation in some local villages.

**Conclusion:**

Traditional knowledge of wild edible plants, owned by Dai, Hani, and Yao people in Jiangcheng County, is rich but at risk of being lost among the young generation. Diversified cultivation of wild edible plants by the local communities could be a solution for the sustainable use of natural resources and to conserve the endangered species in this trans-boundary region.

## Background

Southeast Asia, including Southwest China, is one of the 34 biodiversity hotspots for conservation priorities in the world [1]. The international borders are essential habitat for the survival of many endangered species. Asia contains approximately 82% of the global border hotspots (the richest 5% of border segments) for threatened trans-boundary species, and the distribution of threatened species with trans-boundary ranges is concentrated primarily in Southeast Asia [2]. China shares 1852 km of border with Laos and Vietnam, and there are around 15 cross-border ethnic groups living in this trans-boundary region, with Dai, Hani, and Yao people as three main indigenous groups [3, 4]. Known as a “green pearl” on the Tropic of Cancer in Yunnan, China, Pu’er City is selected as a key conservation area because of its important biodiversity status in Yunnan and even in China [5]. Jiangcheng county, belonging to Pu’er City and located in Southwest China, is the only Chinese county bordered by three countries (China, Laos, and Vietnam). Jiangcheng county was named after its three surrounding rivers and was part of the ancient Ailao Kingdom about 2100 years ago [6]. Geographically, it is situated in the Hengduan Mountain range, lying at the end of Wuliang Mountain with elevation ranging from 317 to 2207 m. It is also a multi-ethnically inhabited region with 25 ethnic groups [7]. All of these make Jiangcheng County a microcosm of the rich bio-cultural kingdom in the trans-boundary regions among China, Laos, and Vietnam.

Wild edible plants, such as vegetables and fruits, play an important role in our daily life. Wild vegetables are favored by more and more people because they have fresh and aromatic taste, rich mineral nutrients, pollution-free growing environment, strong vitality, and high medicinal and human health benefits [8, 9]. Wild edible plants are important in many facets of life for many indigenous and agricultural communities [10–12]. They could provide supplement food, nutrients, medicines, building materials, firewood, dyes, staple, and cash income to native ethnic groups [13–17]. Settled in the low mountain land and faraway from big modern cities, the local people in Jiangcheng County rely a lot on the natural products from the wild. In the past, wild edible plants were mostly self-harvested and consumed as main food substitutes by the local people. Nowadays, wild edible plants are more likely to be sold in the markets for urban citizens and tourists. Thus, the local communities have gathered abundant traditional knowledge from these long-term practices. Previous studies of the wild edible plants in Southwest China focused on providing a list of species [18, 19]. The traditional knowledge associated with the listed species as well as their quantitative inheritance information among different generations were absent from these studies.

Global climatic change poses a huge threat on biodiversity [20], and results in global biodiversity loss through drought and warming [21]. There are an estimated 500,000 species of land plants and a third of all land plants are perhaps at risk of extinction due to habitat loss, fragmentation, and degradation, over-exploitation, invasive species, pollution, and anthropogenic climate change [22]. Current species extinction rates are higher than would be expected and the sixth mass extinction may be under way [23]. Besides, most of the residential ethnic groups in this region depend on the local forest for their livelihood. In addition to the poor economy and excessive collection of wild plants, large-scale rubber and other economic plantation, fragmentation, and a progressively decreased connectivity of forest aggravate the crisis on the sustainable use of the natural resources and the situation of biodiversity conservation in this region is worsening [24–27].

The traditional ecological knowledge, gathered by the indigenous communities in their long interaction with nature, is an important part of human cultural heritage. Much traditional ecological knowledge is under threat and at the verge of disappearing due to environmental changes, livelihood diversification, and the influence of cultural conflicts [28–31]. Meanwhile, the ethnic groups in this region do not have or do not use their own written language, and their traditional knowledge could only be inherited by the next generation through oral communication. Any loss can turn out to be permanent. Thus, the exploration and documentation of the local traditional knowledge of the wild edible plants in this region are of the utmost importance.

The aim of this study was to catalog the traditional knowledge on the wild edible plants used by three trans-boundary ethnic groups in Jiangcheng County, to quantify the inheritance of traditional knowledge, and to provide primary scientific practices for future sustainable utilization and conservation of wild edible plants.

## Methods

### Study area

**Fig. 1:**
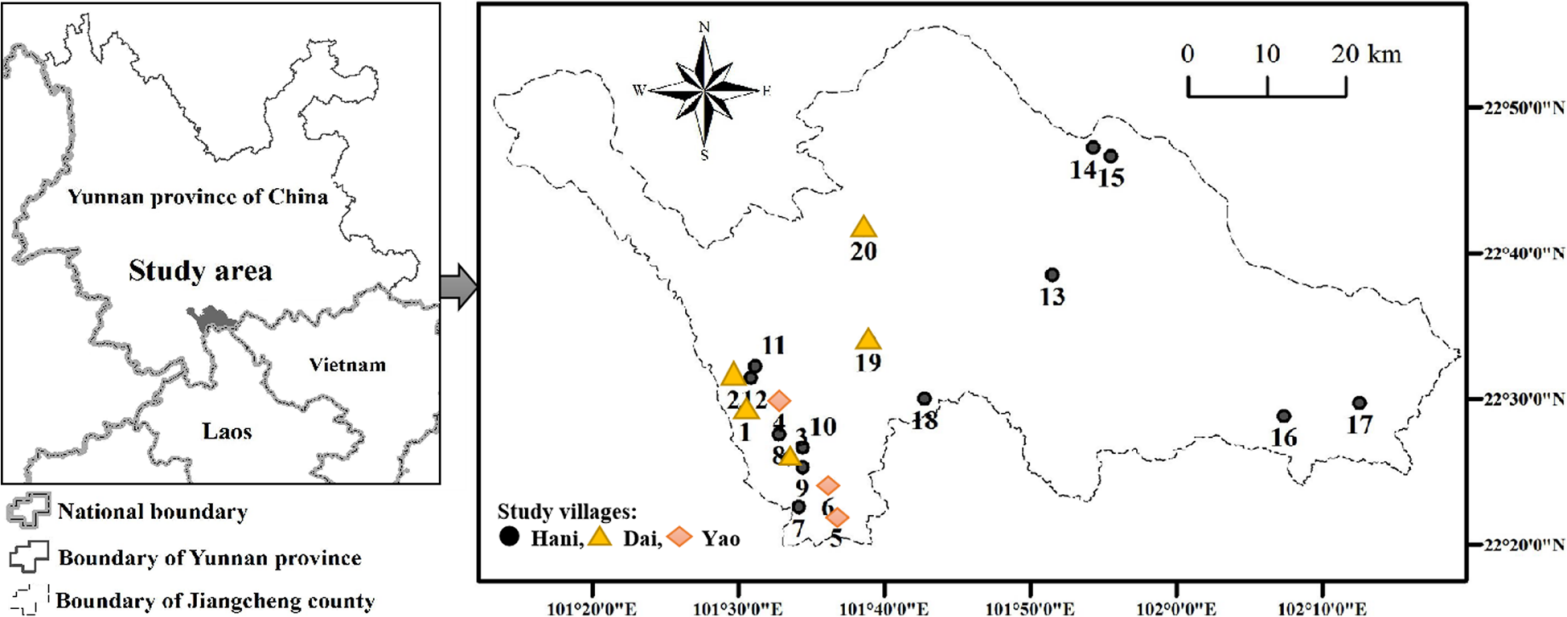
Location of study sites in Jiangcheng Couty, Pu’er City, Southwest, China. Different shapes and colors represented different ethnic villages. Villages no. 1-3 and 19-20 are Dai people’s village named Chenzisanzhai, Mankuan, Mantan, Zhongping, and Shuicheng respectively. Villages no. 4-6 are Yao people’s village named Xiamanqing, Xicaotang, and Xiaomangong respectively. Villages no. 7-18 are Hani people’s village named Huibaohe, Maliqing, Shibajia, Xinjiang, Luoqiya, Nabanhe, Medeng, Gejie, Baga, Basan, Nuna, and Mengkang respectively

Jiangcheng County is bordered by Laos and Vietnam, and its geographical coordinates are between longitudes 101° 14′-102° 19′ east and latitude 22° 20′-22° 36′ north with a subtropical humid climate. Its spring and autumn periods are longer than summer and winter with an average annual rainfall of 2189.3 mm and a comfortable average temperature of around 19.4° [7]. During 2016-2020, ethnobotanical studies on wild edible plants utilized by local people were carried out in 20 villages and nearby markets, distributed at all 6 townships of Jiangcheng County (Fig. 1). Zhengdong, one of township in Jiangcheng, has been recognized with one of the fourth batch of national beautiful and livable townships awards by the Chinese Ministry of Housing and Urban-Rural Development, and as an ecological civilization township by the Yunnan province. Zhengdong town is also an important habitat for around 44 Asian elephants [32]. Nine different villages of Zhengdong township, which are famous for multi-ethnic traditional culture and well-preserved natural forest, were chosen for a detailed study. There is no frost and snow in the whole year, rich in heat resources and fertile land with corn, rice, rubber, tea, coffee, passion flower, nuts, bananas, and mangoes as the main economic crops [7]. Hani, Dai, and Yao people are the three major ethnic minorities that are living at China, Laos, and Vietnam trans-boundary region and have a long tradition and abundant practices of eating wild plants.

### Ethnobotanical survey

**Fig. 2:**
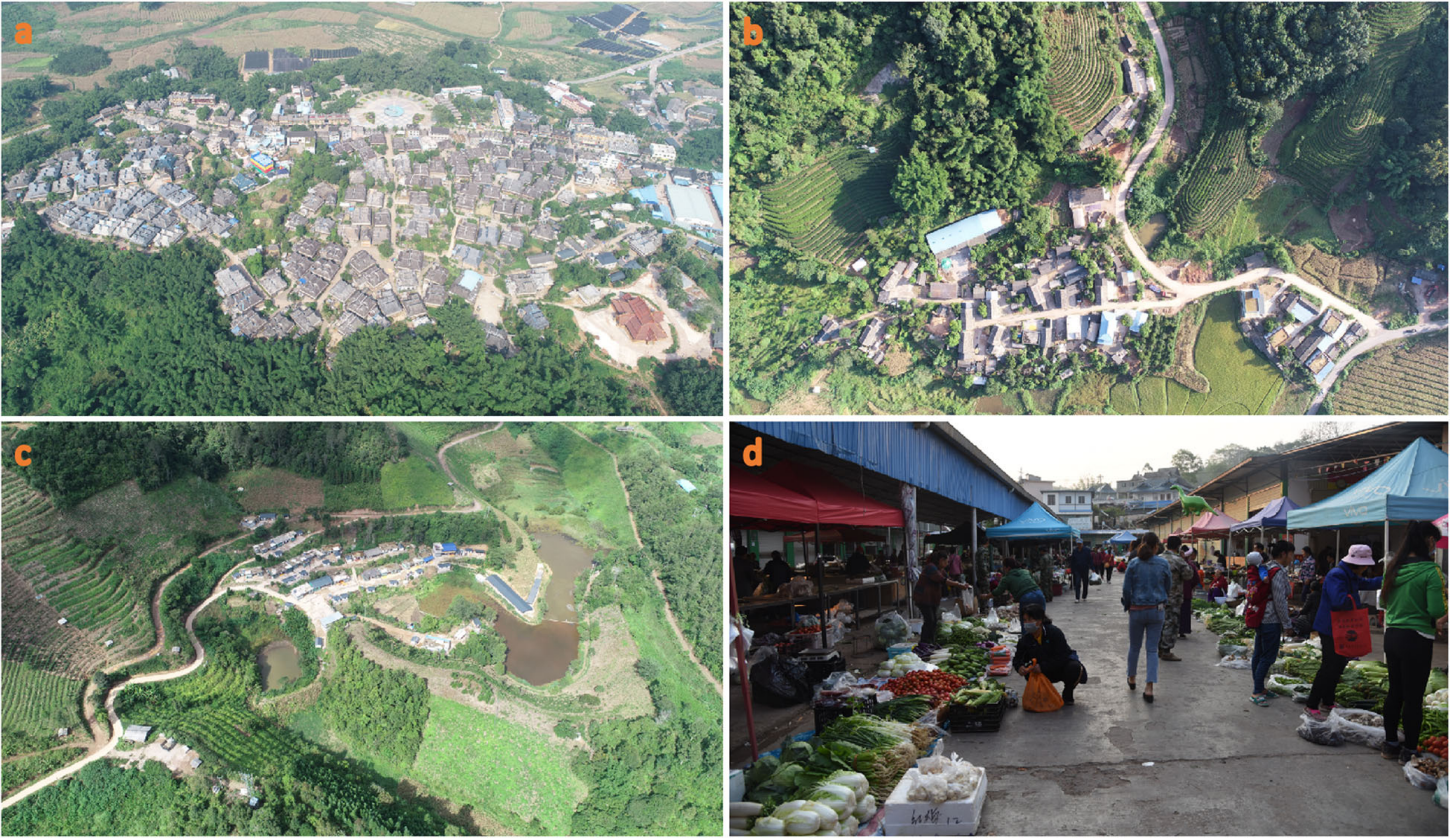
Investigated Dai (**a**), Hani (**b**), Yao (**c**) village, and local market (**d**)

**Fig. 3:**
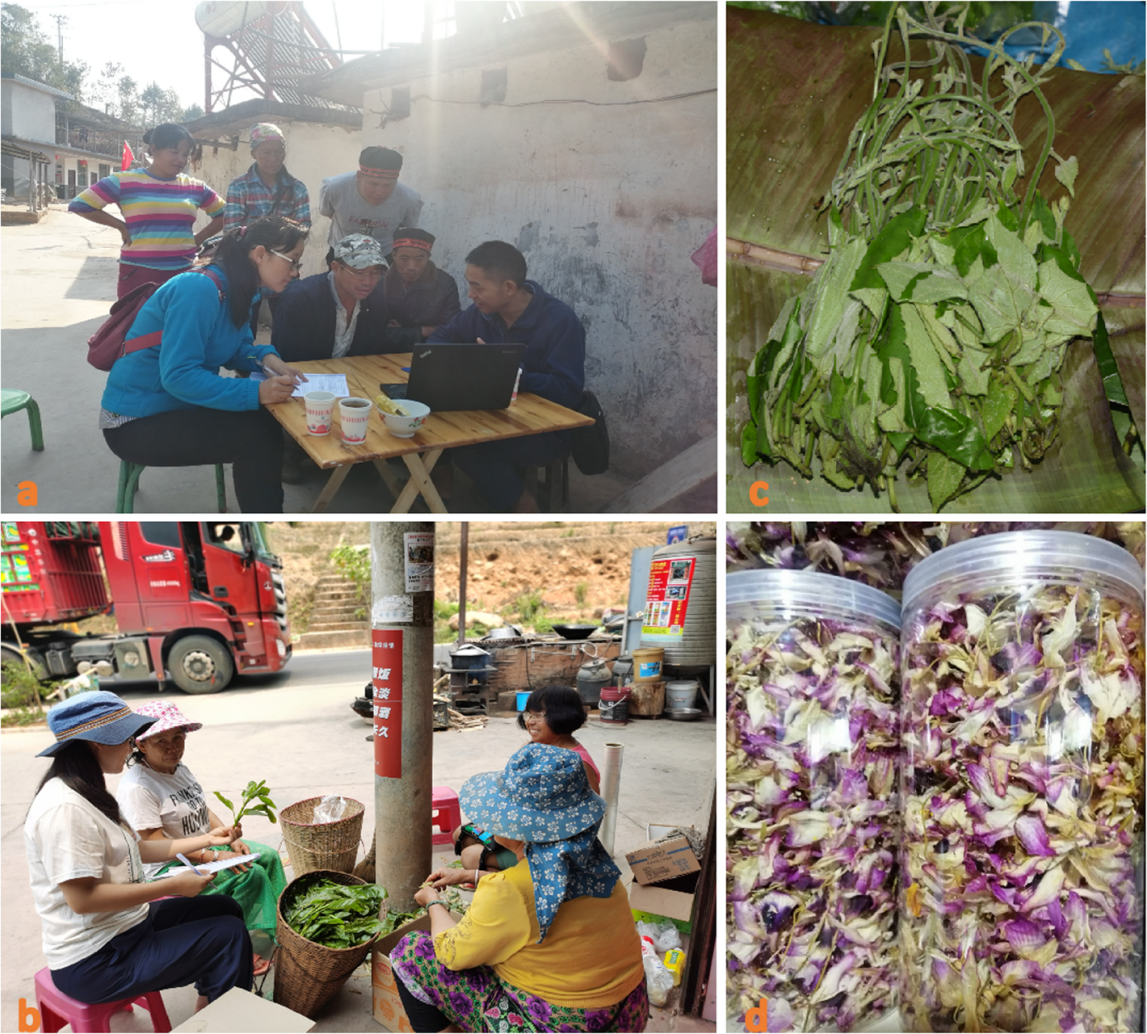
Interviewing the local people (**a**, **b**), informed permissions from the local people have been obtained for the use of the pictures; *Parabaena sagittata* Miers (**c**) and *Dendrobium nobile* Lindl. (**d**) sold at the market

Before ethnobotanical survey in each village, we had a meeting with the village head in which we explained our research objective. Consent from the village head and every interviewed villager was gained and all investigations were conducted following the ethical guidelines of the International Society of Ethnobiology [33]. Ethnobotanical field survey on wild edible plants consumed by three trans-boundary ethnic groups were carried out in 20 villages (13 Hani, 4 Dai, 3 Yao) during different seasons of 2016 to 2020 (Fig. 2). Multiple interdisciplinary methods, including key informant interview, semi-structured interview, and direct observation were used in the survey [34] (Fig. 3). The main informants were introduced by the local village head at first, then recruited haphazardly during house-to-house questioning. A total of 109 informants, including 50 males and 59 females, with ages ranging from 21 to 78 years old, were interviewed. The “5 W + H” questions (i.e., questions concerning what, when, where, who/whom, why, and how the subjects utilize wild edible plants) [34, 35] were used to collect the local name, used parts, usage, preparation methods, function, richness, or availability information of the wild edible plants. The investigations of different markets nearby the village were also conducted to collect the selling and consumption information of wild edible plants in local peoples’ daily life.

Interviews were carried out mainly in Mandarin, although ethnic languages were also used with assistance from local village heads or guides in the study sites. The local names of wild edible plants were recorded by Chinese Pinyin. The collected voucher specimens were identified by the authors with reference to the flora of China and were deposited in the Herbarium of Xishuangbanna Tropical Botanical Garden, Chinese Academy of Sciences (HITBC). We adopted the APG IV system for the taxonomic definition of plant families and species [36]. The uniform nomenclature of plants was given by following the information in The Plant List [37]. The conservation status was recorded by referring to the data from IUCN Red List [38], threatened species list of China’s higher plants [39], and the Subject Database of China Plant [40].

### Data analysis

The use values (UV) of each wild edible plant were calculated to evaluate the relative importance of each plant based on the number of times cited and the number of informants [35, 41]. The formula for UV is UV = (∑ *U*_i_)/*N* [35]. *U*_i_ is the times cited by each informant for a certain wild edible plant, while *N* is the total number of informants. The similarity or dissimilarity of plant species used in each pair of the ethnic communities studied was analyzed with the Jaccard index, JI = *a*/(*a + b + c*), where *a* is the number of species in common; *b* the number of species used only by one specific community, and *c* is the number of species used only in the other community [42]. We used a spreadsheet (Excel) to make the catalog and analysis about the ethnobotanical information of wild edible plants. A uni-variate linear regression analysis was undertaken using R (version 4.0.2) to evaluate the relationship of informants’ age and the number of mentioned wild edible plants. Significant difference was accepted at *P* < 0.05.

## Results and discussion

### Diversity of wild edible plants, life forms, and edible parts in Jiangcheng County

Local people in Jiangcheng County do not have a very strict taxonomy system, and they treat all the plants that are not grown or cultivated in their farm lands or collected from the forest or mountain areas as wild edible plants. A total of 211 wild edible plants, including one feral species *Colocasia esculenta* “Tonoimo”, along with ethnobotanical catalog information such as scientific names, family names, local names, life forms, edible parts, usage and preparations, voucher numbers, cultivation and market status, were recorded (Table 1). The 211 species, belonging to 71 families and 151 genera, account for about 20.2%, 9.0%, and 3.8% of the total families, genera, and species of high plants in Pu’er City [5]. The most frequently used plants are mainly from the family of Poaceae (14 species), Fabaceae (12 species), Lamiaceae (11 species), Zingiberaceae (8 species), Araliaceae (7 species), Amaranthaceae (7 species), and Moraceae (7 species). At genus level, *Dendrocalamus*, *Dioscorea*, *Solanum*, *Amaranthus*, *Amomum*, *Colocasia*, *Dendrobium*, *Ficus*, *Musa*, and *Zanthoxylum* contain 4 to 6 species. There are 29 families and 119 genera that include only one species in the list. This is one of the longest list of local wild edible plants published, and reveals that this is a region of global significance for wild edible plant diversity.

**Table 1:** List of wild edible plants used by Dai, Hani, and Yao people in Jiangcheng County, Pu’er, Southwest, China

Family name	Scientific name	Local name^**a**^	Life form	Used part	Preparation and usage	Voucher number	WC^**b**^	MS^**c**^	UV^**d**^
Acanthaceae	*Dicliptera chinensis* (L.) Juss.	ya ke suo (D), nan lan (Y)	Herb	Tender stem and leaf	Dye material to make red sticky rice	C - 110	W	N	0.39
Acanthaceae	*Thunbergia grandiflora* (Rottl. ex Willd .) Roxb.	za kuo luo kuo (H), lao gua dan (Y)	Woody vine	Flower	Potherb; tea substitute	C - 188	W	N	0.21
Alismataceae	*Limnocharis flava* (L.) Buch.	an mu (H), gai jiu (Y)	Herb	Tender stem and leaf	Potherb	C - 111	W	N	0.19
Alismataceae	*Sagittaria trifolia* L.	ya gan gai (D), de si de luo (H), gai jiu ding (Y)	Herb	Rhizome	Potherb	C - 187	W	N	0.46
Amaranthaceae	*Alternanthera sessilis* (L.) DC.	pa biu (D), e luo er la (H)	Herb	Tender stem and leaf	Salad	C - 077	W	N	0.14
Amaranthaceae	*Amaranthus blitum* L.	pa hong kei (D), mo tuo qi pu (H), jia gai hen (Y)	Herb	Tender stem and leaf	Potherb	C - 009	W	Y	1
Amaranthaceae	*Amaranthus spinosus* L.	pa hong nan (D), gai hen lu (Y)	Herb	Tender stem and leaf	Potherb	C - 007	W	Y	0.92
Amaranthaceae	*Amaranthus tricolor* L.	pa hong (D), mo tuo qi pu (H), gai heng (Y)	Herb	Tender stem and leaf	Potherb	C - 008	W, C	Y	0.97
Amaranthaceae	*Amaranthus viridis* L.	pa hong kei (D), mo tuo qi pu (H), gai hen dong (Y)	Herb	Tender stem and leaf	Potherb	C - 105	W	Y	0.99
Amaranthaceae	*Chenopodium album* L.	pa hong ge (D), nuo za (H), ma lan gai (Y)	Herb	Tender stem and leaf	Potherb	C - 103	W	Y	0.95
Amaranthaceae	*Chenopodium ficifolium* Smith	pa hong ge (D), nuo za (H), ma lan gai (Y)	Herb	Tender stem and leaf	Potherb	C - 031	W	Y	0.83
Anacardiaceae	*Choerospondias axillaris* (Roxb.) B.L. Burtt & A.W. Hill	mang men (D), biu la yang (Y)	Tree	Fruit	Seasonal fruit; sour condiment	C - 038	W	N	0.61
Anacardiaceae	*Mangifera siamensis* Warbg. ex Craib	mong (D), mao mao si (H), dang lao gang (Y)	Tree	Fruit	Seasonal fruit; salad	C - 019	W	Y	1
Anacardiaceae	*Mangifera sylvatica* Roxb.	mong wo (D), dong lao miu (Y)	Tree	Fruit	Seasonal fruit; salad	C - 195	W	N	0.94
Anacardiaceae	*Rhus chinensis* Mill.	po (D), sheng bao che (H), ga fa biu (Y)	Tree	Fruit	Sour condiment	C - 054	W	Y	0.88
Anacardiaceae	*Spondias pinnata* (L. F.) Kurz	guo (D), pi luo si (H), **ga li le biu** (Y)	Tree	Fruit; tender stem and leaf	Potherb; sour condiment; liquor brewing	C - 029	W, C	Y	0.94
Apiaceae	*Centella asiatica* (L.) Urban	pa nuo (D), me kuo luo guo (H), da (Y)	Herb	Tender stem and leaf	Potherb; salad	C - 011	W	Y	0.96
Apiaceae	*Eryngium foetidum* L.	pa bong men pon (D), **yan xu** (H), long bia jing (Y)	Herb	Young leaf	Spices	C - 153	W, C	Y	0.94
Apiaceae	*Heracleum bivittatum* H. de Boissieu	mu ling wong (Y)	Herb	Root	Tonic soup	C - 177	W	N	0.1
Apiaceae	*Oenanthe javanica* (Blume) DC.	wo guo wo luo (H), gai weng (Y)	Herb	Tender stem and leaf	Potherb	C - 194	W	Y	0.64
Apocynaceae	*Amalocalyx microlobus* Pierre	xin ha (D), mu nuo qi cha (H), long rui biu (Y)	Herbaceous vine	Fruit	Salad	C - 116	W	Y	0.98
Apocynaceae	*Dregea volubilis* (Linnaeus f.) Bentham ex J. D. Hooker	pa men (D), ku teng (H), ma ying mei (Y)	Woody vine	Flower; tender stem and leaf	Potherb	C - 193	W, C	Y	0.94
Apocynaceae	*Urceola rosea* (Hooker & Arnott) D. J. Middleton	song long (D)	Woody vine	Tender stem and leaf	Sour condiment	C - 043	W	N	0.27
Araceae	*Amorphophallus krausei* Engler	yi luo (D), gei ang (H), gui (Y)	Herb	Rhizome	Potherb	C - 156	W	Y	0.96
Araceae	*Colocasia esculenta* (L.) Schott.	bo rui (D), biu o (H), hou min deng (Y)	Herb	Rhizome; flower; petiole	Potherb	C - 051	C	Y	0.98
Araceae	*Colocasia esculenta* “Tonoimo”	pei wan (D), biu wo (H), hou gun di (Y)	Herb	Rhizome; petiole	Potherb	C - 180	C	Y	0.83
Araceae	*Colocasia fallax* Schott	bo rui (D), biu o (H), hou dan (Y)	Herb	Rhizome; petiole	Potherb	C - 068	W	N	0.77
Araceae	*Colocasia gigantea* (Blume) Hook. f.	bo rui (D), you ti (H), hou bu (Y)	Herb	Petiole	Potherb	C - 084	W, C	Y	0.9
Araceae	*Lasia spinosa* (L.) Thwaites	pa bo nan (D), **ci bao cai** (H), gian dei ai (Y)	Herb	Tender stem and leaf	Potherb	C - 131	W	Y	0.93
Araliaceae	*Aralia armata* (Wall.) Seem.	pa dan (D), **ci bao cai** (H), dong gong yang (Y)	Tree	Tender stem and leaf	Potherb	C - 055	W	Y	0.61
Araliaceae	*Brassaiopsis glomerulata* (Blume) Regel	guo dan (D), ta bi ta la (H), dong gong lu (Y)	Tree	Tender stem and leaf	Potherb	C - 152	W	N	0.06
Araliaceae	*Eleutherococcus trifoliatus* (L.) S.Y. Hu	ha bing (D), wu jiao fu (H), **ci wu jia** (Y)	Shrub	Tender stem and leaf	Potherb; salad	C - 017	W, C	Y	0.98
Araliaceae	*Macropanax dispermus* (Blume) Kuntze	a sa ding (Y)	Tree	Tender stem and leaf	Potherb	C - 089	W	Y	0.15
Araliaceae	*Panax japonicus* (T. Nees) C. A. Meyer	**san qi** (H), dang sa wang (Y)	Herb	Root	Liquor brewing; tonic soup	Z - 9429	W	Y	0.15
Araliaceae	*Panax zingiberensis* C.Y. Wu & K.M. Feng	**san qi** (H), dang sa (Y)	Herb	Root	Liquor brewing; tonic soup	T - 44035	W	Y	0.14
Araliaceae	*Trevesia palmata* (Roxburgh ex Lindley) Visiani	pa dan (D), dong gong lu (Y)	Tree	Tender stem and leaf	Potherb	C - 070	W	Y	0.17
Arecaceae	*Calamus henryanus* Becc.	wai (D), ge lan bie (Y)	Herbaceous vine	Tender stem heart	Potherb	C - 164	W	N	0.05
Arecaceae	*Caryota maxima* Blume ex Martius	guo zhu (D), **dong zong** (H), dei din (Y)	Tree	Tender stem heart	Potherb	C - 173	W	Y	0.86
Arecaceae	*Caryota obtusa* Griffith	guo bang (D), la wo ba ma (H), dei din (Y)	Tree	Tender stem heart	Potherb	C - 132	W	Y	0.49
Arecaceae	*Livistona saribus* (Lour.) Merr. ex A. Chev.	ma guo (D), guo (Y)	Tree	Fruit	Potherb	C - 157	W	Y	0.18
Asparagaceae	*Polygonatum cirrhifolium* (Wallich) Royle	**ma wei gen** (D), huo pi da guo (H), bing du jiang (Y)	Herb	Root	Tonic soup	C - 013	W, C	Y	0.41
Asparagaceae	*Polygonatum kingianum* Coll. et Hemsl.	**ma wei gen** (D), huo pi da guo (H), bing du lu (Y)	Herb	Root	Tonic soup	C - 134	W, C	Y	0.44
Asteraceae	*Arctium lappa* L.	**niu ban zi gen** (H), **niu bang zi geng** (Y)	Herb	Root	Tonic soup	C - 192	C	Y	0.27
Asteraceae	*Bidens pilosa* L.	ya dong long (D), za qie mo (H), ma zhan (Y)	Herb	Tender stem and leaf	Potherb	C - 085	W	N	0.76
Asteraceae	*Crassocephalum crepidioides* (Benth.) S. Moore	ya ge la (D), ming guo cao (H), dong ma gun (Y)	Herb	Tender stem and leaf	Potherb	C - 006	W	Y	0.93
Asteraceae	*Elephantopus scaber* L.	ya din dian (D), bo ga ga sa (H), ma bie min (Y)	Herb	Root	Tonic soup	C - 140	W	Y	0.56
Asteraceae	*Gynura divaricata* (L.) DC.	pa bong bang (D)	Herb	Tender stem and leaf	Potherb	C - 170	W, C	N	0.06
Asteraceae	*Sonchus oleraceus* L.	**ku mei cai** (Y)	Herb	Young leaf	Potherb	C - 080	W	N	0.11
Athyriaceae	*Diplazium esculentum* (Retz.) Sm.	guo gun (D), de pi (H), jiao gai (Y)	Herb	Tender stem and leaf	Potherb	C - 049	W	Y	0.96
Athyriaceae	*Diplazium esculentum* var. *pubescens* Tardeiu et C. Chr.	pa guo (D), da guo guo me (H), jiao gai lu (Y)	Herb	Tender stem and leaf	Potherb	C - 086	W	N	0.36
Balsaminaceae	*Impatiens mengtszeana* J. D. Hooker	ya die da (D), la you (H), gei gai (Y)	Herb	Tender stem and leaf	Potherb	C - 088	W	N	0.24
Basellaceae	*Anredera cordifolia* (Tenore) Steenis	pa bang (D), mei dang sha (Y)	Herbaceous vine	Tender stem and leaf	Potherb	C - 041	W, C	Y	0.21
Begoniaceae	*Begonia augustinei* Hemsl.	pa gan song (D), pa che e ge (H), a ei (Y)	Herb	Tender stem and leaf; stem	Potherb; sour condiment	C - 214	W	N	0.76
Begoniaceae	*Begonia longifolia* Blume	pa gan song (D), yao me che ge (H), a ei dan (Y)	Herb	Tender stem and leaf; stem	Potherb; sour condiment	C - 087	W	N	0.68
Begoniaceae	*Begonia silletensis* subsp. *mengyangensis* Tebbitt & K. Y. Guan	pa gan song (D), yao me che ge (H), a ei lu (Y)	Herb	Tender stem and leaf; stem	Potherb; sour condiment	C - 167	W	N	0.72
Bignoniaceae	*Markhamia stipulata* (Wall.) Seem.	lao gei (D), xie xie a yi (H), mao dei fan (Y)	Tree	Flower; fruit	Potherb	C - 072	W	N	0.59
Bignoniaceae	*Markhamia stipulata* var. *kerrii* Sprague	lao gei (D), xie xie a yi (H), mao dei fan (Y)	Tree	Flower; fruit	Potherb	C - 138	W	N	0.6
Bignoniaceae	*Mayodendron igneum* (Kurz) Kurz	lao bie (D), guo te guo me a ye (H), nia long fan (Y)	Tree	Flower	Potherb	C - 027	W	Y	0.99
Bignoniaceae	*Oroxylum indicum* (L.) Bentham ex Kurz	lin deng a (D), bu gu bu lie (H), dao din (Y)	Tree	Flower; fruit; tender stem and leaf	Potherb; salad	C - 189	W, C	Y	0.97
Boraginaceae	*Trichodesma calycosum* Coll. et Hemsl.	mao duo da (D), deng long fang (Y)	Shrub	Flower	Potherb	C - 109	W	Y	0.39
Brassicaceae	*Cardamine hirsuta* L.	wang ye gai (Y)	Herb	Tender stem and leaf	Potherb	C - 097	W	N	0.19
Brassicaceae	*Nasturtium officinale* R. Br.	pa nan (D), ye qing cai (Y)	Herb	Tender stem and leaf	Potherb	C - 100	W	Y	0.71
Brassicaceae	*Rorippa indica* (L.) Hiern	pa ya guo mu (D)	Herb	Tender stem and leaf	Potherb	C - 081	W	N	0.28
Cabombaceae	*Brasenia schreberi* J.F. Gmel.	pong (D), xin ga la mo (H)	Herb	Young leaf	Potherb	W - 81210	W	N	0.25
Campanulaceae	*Codonopsis javanica* (Blume) Hook.f. & Thomson	ma gong gui (D), ya li bo me (H), nian bao biu (Y)	Herb	Root; tender stem and leaf	Tonic soup; potherb	C - 191	W	Y	0.66
Campanulaceae	*Lobelia nummularia* Lam.	dong ke (D), **di si liu** (H), long nan ma (Y)	Herb	Tender stem and leaf	Tonic soup	C - 091	W	Y	0.94
Capparaceae	*Crateva unilocularis* Buchanan-Hamilton	pa gong (D), wo ni kuo tuo luo (H), zhai niao gai (Y)	Tree	Tender stem and leaf	Salad	C - 005	W, C	Y	0.95
Caricaceae	*Carica papaya* L.	gui su bao (D), me mao si (H), jin gua biu (Y)	Tree	Fruit	Salad; potherb	C - 016	W, C	Y	0.94
Caryophyllaceae	*Brachystemma calycinum* D. Don	ya ying ren (D), pi si li guo (H), du sha ma (Y)	Herb	Root	Tonic soup	C - 101	W	Y	0.44
Clusiaceae	*Garcinia cowa* Roxb.	guo da (D), yi ka bu duo si (H), ke diu biu (Y)	Tree	Fruit	Seasonal fruit	C - 213	W	N	0.44
Commelinaceae	*Commelina communis* L.	ya song (D), wa you (H), dang dai (Y)	Herb	Tender stem and leaf	Potherb	C - 033	W	N	0.18
Commelinaceae	*Streptolirion volubile* Edgew.	bi (D), dang dai lu (Y)	Herbaceous vine	Inflorescence	Potherb	C - 210	W	N	0.11
Costaceae	*Cheilocostus speciosus* (J.Koenig) C.D.Specht	mai eng (D), mi jie (H), mu long dong bia (Y)	Herb	Young shoot	Potherb	C - 073	W	N	0.09
Cucurbitaceae	*Coccinia grandis* (L.) Voigt	ma dian lu (D), ni qi pi li po luo (H)	Herbaceous vine	Fruit	Potherb	C - 166	C	N	0.47
Cucurbitaceae	*Cucumis hystrix* Chakr.	dian song (D), a yao shuo kuo (H), lu gua biu (Y)	Herbaceous vine	Fruit	Salad	C - 215	W	Y	0.81
Cucurbitaceae	*Gynostemma pentaphyllum* (Thunb.) Makino	lei ya zha kuo (H), gai ya (Y)	Herbaceous vine	Tender stem and leaf	Potherb	C - 039	W	Y	0.49
Cucurbitaceae	*Hodgsonia heteroclita* (Roxb.) Hook. f. et Thomson	man mo (D), er pi duo luo (H), geng gua mei biu (Y)	Herbaceous vine	Seed	Nut	C - 147	W	Y	0.7
Cucurbitaceae	*Momordica subangulata* Blume	huai (D), ku gua (H), lu gua yong biu (Y)	Herbaceous vine	Fruit	Potherb	C - 118	W	N	0.71
Cycadaceae	*Cycas pectinata* Buchanan-Hamilton	guo gu (D), da gu (H)	Tree	Young leaf	Potherb	C - 092	W, C	N	0.12
Dennstaedtiaceae	*Pteridium aquilinum* var. *latiusculum* (Desv.)Underw.ex Heller	guo gun (D), da gu guo yao (H), jiao (Y)	Herb	Tender stem and leaf	Potherb	C - 034	W	Y	0.79
Dennstaedtiaceae	*Pteridium revolutum* (Blume) Nakai	guo gun (D), da gu guo ma (H), jiao lu (Y)	Herb	Tender stem and leaf	Potherb	C - 178	W	Y	0.74
Dilleniaceae	*Dillenia indica* L.	san (D), si pi luo me (H), bia huo biu (Y)	Tree	Fruit	Salad	C - 106	W	N	0.83
Dioscoreaceae	*Dioscorea alata* L.	man bo (D), cao bao me (H), dei ling liu (Y)	Herbaceous vine	Rhizome	Potherb	C - 130	W	Y	0.75
Dioscoreaceae	*Dioscorea bulbifera* L.	yi bao (D), ka la si (H), lu gong lai biu (Y)	Herbaceous vine	Rhizome	Potherb	C - 184	W	N	0.28
Dioscoreaceae	*Dioscorea esculenta* var. *spinosa* (Roxb.ex Prain et Burkill) R.Knuth	man nan (D), me ci (H), duo bing liu (Y)	Herbaceous vine	Rhizome	Potherb	C - 165	C	Y	0.84
Dioscoreaceae	*Dioscorea fordii* Prain & Burkill	man nei (D), me ka (H), le lan gun (Y)	Herbaceous vine	Rhizome	Potherb	C - 129	W	Y	0.62
Dioscoreaceae	*Dioscorea pentaphylla* L.	yi bao (D), de de (H), dui yi bao (Y)	Herbaceous vine	Rhizome	Potherb	C - 115	W	N	0.51
Dioscoreaceae	*Dioscorea yunnanensis* Prain & Burkill	man nei (D), a niu (H), lui wie (Y)	Herbaceous vine	Rhizome	Potherb	C - 209	W	N	0.45
Elaeagnaceae	*Elaeagnus conferta* Roxb.	luan (D), mu long ning biu (Y)	Woody vine	Fruit	Salad	C - 082	W, C	Y	0.96
Elaeocarpaceae	*Elaeocarpus austroyunnanensis* Hu	dao du biu (Y)	Tree	Fruit	Seasonal fruit	C - 146	W	Y	0.45
Ericaceae	*Vaccinium exaristatum* Kurz	ma di (D), a me te li (H), ge die yang (Y)	Tree	Fruit; tender stem and leaf	Seasonal fruit; potherb	C - 154	W	N	0.72
Ericaceae	*Vaccinium harmandianum* Dop	a mu te lie (H), ge die yang (Y)	Tree	Fruit; flower; tender stem and leaf	Potherb; seasonal fruit	C - 117	W	N	0.53
Erythropalaceae	*Erythropalum scandens* Blume	gai yang (Y)	Woody vine	Tender stem and leaf	Potherb	C - 197	W	Y	0.13
Euphorbiaceae	*Euphorbia neriifolia* L.	k li ma (D), si lei bo dong (H), long gua (Y)	Tree	Stem	Tonic soup	C - 202	W, C	N	0.42
Euphorbiaceae	*Manihot esculenta* Crantz	men niu (D), la bi mu (H), jian lui (Y)	Shrub	Rhizome; tender stem and leaf	Potherb	C - 018	W, C	Y	0.83
Fabaceae	*Acacia concinna* (Willd.) DC.	song bai (D)	Woody vine	Tender stem and leaf	Potherb; sour condiment	C - 150	W, C	N	0.23
Fabaceae	*Acacia pennata* (L.) Willd.	pa ge da (D), tao pu (H), miao tei (Y)	Woody vine	Tender stem and leaf	Potherb	C - 199	W, C	Y	0.99
Fabaceae	*Afgekia filipes* (Dunn) R. Geesink	luo pai wang (D), mie yi (H), mei bie fang (Y)	Woody vine	Flower	Potherb	C - 045	W	Y	0.94
Fabaceae	*Bauhinia variegata* var. *candida* (Roxb.) Voigt	luo ke xiu (D), qie ti er yi (H), yang bian fang (Y)	Tree	Flower	Potherb	C - 025	W	Y	0.99
Fabaceae	*Cajanus cajan* (L.) Millsp.	tu ye (D), ne qie ke lie (H), de bei nie jian (Y)	Shrub	Seed	Potherb	C - 159	W	N	0.37
Fabaceae	*Crotalaria pallida* Ait.	ma chong chan (D), za kuo luo kuo (H), ge ling lu (Y)	Herb	Flower	Potherb	C - 037	W	N	0.2
Fabaceae	*Erythrina subumbrans* (Hassk.) Merr.	guo dong (D), ke xie a yi (H), mu long gian (Y)	Tree	Flower	Potherb	C - 208	W	N	0.28
Fabaceae	*Millettia pachycarpa* Benth.	shu nan yi (H), lai mei (Y)	Woody vine	Flower	Potherb	C - 056	W	N	0.02
Fabaceae	*Mucuna macrocarpa* Wall.	ke tuo (D), ne qi a yi (H), gua la lu (Y)	Woody vine	Flower	Potherb	C - 145	W	N	0.39
Fabaceae	*Mucuna pruriens* (L.) DC.	ne qi a yi (H), mei lan (Y)	Herbaceous vine	Flower	Potherb	C - 046	W	N	0.27
Fabaceae	*Pachyrhizus erosus* (L.) Urb.	huo guo den (D), di le bu (H), ni ge ba (Y)	Herbaceous vine	Rhizome	Seasonal fruit; potherb	C - 162	C	Y	1
Fabaceae	*Pueraria montana* (Loureiro) Merrill	ke bie (D), qi guo (H), mei bie (Y)	Herbaceous vine	Root	Potherb	C - 050	W, C	Y	0.9
Gnetaceae	*Gnetum montanum* Markgraf	ke mei (D), pai li guo (H), ge mai mei(Y)	Woody vine	Seed	Nut	C - 067	W	Y	0.64
Hydroleaceae	*Hydrolea zeylanica* (L.) Vahl	pa bu yin (D), huang shang dang (H)	Herb	Tender stem and leaf	Potherb	C - 206	W	Y	0.58
Lamiaceae	*Clerodendrum chinense* var. *simplex* (Moldenke) S.L. Chen	bei bing (D), de ga pa me (H), gong mie gian (Y)	Shrub	Young leaf	Potherb; Salad	C - 065	W	N	0.47
Lamiaceae	*Clerodendrum japonicum* (Thunb.) Sweet	bei bing (D), de ga pa me (H)	Shrub	Flower	Salad; potherb; tonic soup	C - 137	W, C	Y	0.34
Lamiaceae	*Elsholtzia blanda* (Bentham) Bentham	ya you man nuai (D), lu gu me (H), dan mie (Y)	Herb	Tender stem and leaf	Tea substitute	C - 127	W	N	0.45
Lamiaceae	*Elsholtzia kachinensis* Prain	pa leng (D), mi ge li guo (H), ma de dan (Y)	Herb	Tender stem and leaf	Salad; potherb	C - 075	W, C	Y	1
Lamiaceae	*Elsholtzia rugulosa* Hemsley	pu la huo (D), ni ke ni ne (H), bu da za (Y)	Shrub	Tender stem and leaf	Tea substitute	C - 112	W	N	0.78
Lamiaceae	*Gmelina arborea* Roxb.	luo suo (D), a yi huo si (H)	Tree	Flower	Dye material to make brown sticky rice	C - 171	W, C	N	0.36
Lamiaceae	*Leonurus japonicus* Houttuyn	**yi mu cao** (H, Y)	Herb	Tender stem and leaf	Potherb; tonic soup	C - 205	W	Y	0.17
Lamiaceae	*Mentha canadensis* L.	zha hong leng (D), lao su ba kuo (H)	Herb	Tender stem and leaf	Spices	C - 211	W	N	0.4
Lamiaceae	*Mentha crispata* Schrader ex Willdenow	huo leng (D), luo ci bo kuo (H), ma du lu (Y)	Herb	Tender stem and leaf	Spices	C - 074	C	Y	1
Lamiaceae	*Ocimum basilicum* L.	gong guo (D), yi ge sa lan (H), **jun gai** (Y)	Herb	Tender stem and leaf	Spices	C - 079	W, C	Y	0.95
Lamiaceae	*Ocimum basilicum* var. *pilosum* (Willd.) Benth.	gan guo ten (D), ei se sa la (H)	Herb	Tender stem and leaf	Spices	C - 114	W	N	0.35
Lauraceae	*Cinnamomum parthenoxylon* (Jack) Meisner	mai dang hu (D), shi xiao si (H), ge long jiang (Y)	Tree	Fruit	Spices	C - 094	W, C	N	0.14
Lauraceae	*Cinnamomum subavenium* Miq.	guo bai san (D), cuo pi cuo guo luo (H), ge long jiang(Y)	Tree	Bark	Spices	C - 124	W	N	0.29
Lauraceae	*Litsea cubeba* (Lour.) Pers.	guo sai kai teng (D), si xiao si (H), ge zhang mu jiang (Y)	Tree	Fruit	Spices	C - 095	W	Y	0.94
Lygodiaceae	*Lygodium salicifolium* Presl	pa guo (D), da guo (H), bu gu jiao (Y)	Herbaceous vine	Tender stem and leaf	Potherb	C - 158	W	N	0.17
Malvaceae	*Bombax ceiba* L.	guan niu (D), yi ka bu duo si (H), **mu mian yang** (Y)	Tree	Seed; flower	Nut; Potherb	C - 201	W	N	0.2
Melanthiaceae	*Paris polyphylla var. yunnanensis* (Franchet) Handel-Mazzetti	**chong le** (D), **chong lou** (H, Y)	Herb	Root	Tonic soup	C - 172	W, C	Y	0.53
Melastomataceae	*Melastoma malabathricum* L.	guo gao (D), bi bi nan nan (H), bu zhang yang (Y)	Shrub	Fruit	Seasonal fruit	C - 048	W	N	0.67
Meliaceae	*Toona sinensis* (A. Juss.) Roem.	fu mei rong (D), ye bu (H), **xiang chun** (Y)	Tree	Tender stem and leaf	Potherb; salad	C - 169	W, C	Y	0.94
Menispermaceae	*Parabaena sagittata* Miers	pan nan (D), xin ga la mo (H), gai mei (Y)	Herbaceous vine	Tender stem and leaf	Potherb	C - 064	W	Y	1
Moraceae	*Artocarpus heterophyllus* Lam.	ma mi (D), mi duo luo (H), long di biu (Y)	Tree	Fruit	Seasonal fruit	C - 161	C	Y	0.99
Moraceae	*Broussonetia papyrifera* (Linnaeus) L'Heritier ex Ventenat	guo sha (D), na sha er zi (H), rou yang (Y)	Tree	Flower; tender stem and leaf; fruit	Potherb	C - 212	W	N	0.33
Moraceae	*Ficus auriculata* Lour.	pa wa (D), na sha er zi (H), long o biu (Y)	Tree	Tender stem and leaf; fruit	Potherb; seasonal fruit	C - 071	W, C	Y	0.93
Moraceae	*Ficus racemosa* L.	guo de (D), mo luo si (H), ge long bie biu (Y)	Tree	Tender stem and leaf; fruit	Potherb	C - 144	W	N	0.31
Moraceae	*Ficus semicordata* Buch.-Ham. ex J. E. Smith	nua (D), si guo si (H), bu luo biu (Y)	Tree	Fruit	Seasonal fruit	C - 057	W	N	0.9
Moraceae	*Ficus virens* Aiton	pa luo (D), nuo na zi (H), ge bpong yang (Y)	Tree	Tender stem and leaf	Potherb	C - 198	W, C	Y	0.93
Moraceae	*Morus alba* L.	mang men (D), shuo zi a bu (H), meng shou nan yang (Y)	Tree	Fruit; tender stem and leaf	Seasonal fruit; Potherb; liquor brewing	C - 002	W, C	Y	0.93
Musaceae	*Ensete glaucum* (Roxb.) Cheesm.	a pa duo gei (H), di bo (Y)	Herb	Tender stem heart	Potherb	C - 076	W, C	Y	0.61
Musaceae	*Musa acuminata* Colla	bi (D), an pe (H), di ban (Y)	Herb	Flower; tender stem heart	Potherb	C - 069	W	Y	0.9
Musaceae	*Musa basjoo* Siebold & Zuccarini	gui he (D), a si (H), biao diu (Y)	Herb	Fruit; flower; tender stem heart	Seasonal fruit; potherb	C - 204	C	Y	1
Musaceae	*Musa itinerans* Cheesman	bi (D), an ne (H), diu di (Y)	Herb	Flower; tender stem heart	Potherb	C - 136	W	Y	0.99
Musaceae	*Musa yunnanensis* Hakkinen & H. Wang	bi (D), an pe (H), di bu (Y)	Herb	Flower; tender stem heart	Potherb	C - 059	W	Y	0.89
Orchidaceae	*Anthogonium gracile* Lindl.	pa lai bo (D), guo si guo nuo (H), xiao bai ji (Y)	Herb	Rhizome	Tonic soup	C - 183	W, C	Y	0.35
Orchidaceae	*Dendrobium crepidatum* Lindl. ex Paxton	luan nan gai (D), **huang cao** (H), me dao ying (Y)	Herb	Stem; flower	Tea substitute; liquor brewing; tonic soup	C - 174	W, C	Y	0.51
Orchidaceae	*Dendrobium cucullatum* R. Br. ex Lindl.	luan nan gai (D), **huang cao** (H), me dao lu (Y)	Herb	Stem; flower	Tea substitute; liquor brewing; tonic soup	C - 123	W, C	Y	0.52
Orchidaceae	*Dendrobium devonianum* Paxton	luan nan gai (D), **huang cao** (H), me dao dang (Y)	Herb	Stem	Tea substitute; liquor brewing; tonic soup	C - 122	W, C	Y	0.51
Orchidaceae	*Dendrobium nobile* Lindl.	luan nan gai (D), **huang cao** (H), me dao lu (Y)	Herb	Stem; flower	Tea substitute; liquor brewing; tonic soup	C - 128	W, C	Y	0.52
Oxalidaceae	*Oxalis corniculata* L.	pa yuan (D), an ni ze che (H), ma bian (Y)	Herb	Young leaf	Potherb	C - 014	W	N	0.5
Passifloraceae	*Adenia cardiophylla* (Mast.) Engl.	ma ti ga (D), a guo cha ba (H)	Herbaceous vine	Tender stem and leaf	Salad	C - 148	W	N	0.28
Passifloraceae	*Passiflora caerulea* Linnaeus	nuo wang wai (D), lao fan guo (H), luo han biu (Y)	Herbaceous vine	Tender stem and leaf; fruit	Potherb; seasonal fruit	C - 179	W, C	Y	1
Phyllanthaceae	*Antidesma acidum* Retz.	guo hua (D), pa che e si (H)	Tree	Tender stem and leaf; fruit	Salad; seasonal fruit	C - 149	W	N	0.31
Phyllanthaceae	*Baccaurea ramiflora* Loureiro	fai (D), si shuo si (H), men lai biu (Y)	Tree	Fruit	Seasonal fruit; salad	C - 060	W, C	Y	0.99
Phyllanthaceae	*Bischofia javanica* Blume	gao (D), si pu ge lie (H), ge ben jian (Y)	Tree	Tender stem and leaf; fruit	Potherb; sour condiment	C - 058	W	N	0.81
Phyllanthaceae	*Glochidion sphaerogynum* (Müll. Arg.) Kurz	guan lei (D), wa lu jie pi (H)	Tree	Tender stem and leaf	Potherb	C - 030	W	N	0.52
Phyllanthaceae	*Phyllanthus emblica* L.	ma bo (D), si cuo si (H), nia gong biu (Y)	Tree	Fruit; bark	Seasonal fruit; salad; liquor brewing	C - 200	W	Y	0.99
Phyllanthaceae	*Sauropus androgynus* (L.) Merr.	pa wan (D), qi du (H), **shu tian cai** (Y)	Shrub	Tender stem and leaf	Potherb	C - 004	C	Y	0.96
Phytolaccaceae	*Phytolacca acinosa* Roxb.	ni zhuo mo (H)	Herb	Tender stem and leaf	Potherb	C - 104	W	N	0.13
Piperaceae	*Piper flaviflorum* C. DC.	ke pian (D), ke duo ye (H), lao mei (Y)	Woody vine	Stem; tender stem and leaf	Spices; potherb	C - 096	W	N	0.12
Piperaceae	*Piper sarmentosum* Roxb.	pa die (D)	Herb	Tender stem and leaf	Potherb	C - 098	W, C	N	0.26
Plantaginaceae	*Limnophila rugosa* (Roth) Merrill	**shui ba guo** (H), lan ba bo he (Y)	Herb	Tender stem and leaf	Spices	C - 190	W	N	0.25
Plantaginaceae	*Plantago asiatica* L.	ya yin ren (D), a mei ye (H), ma dei gan (Y)	Herb	Tender stem and leaf	Potherb	C - 023	W	Y	0.36
Poaceae	*Bambusa lapidea* McClure	nuo piu (D), wo bu (H), lao jing (Y)	Herb	Bamboo	Potherb	C - 175	W, C	N	0.51
Poaceae	*Cymbopogon citratus* (D. C.) Stapf	sa kai (D), po pi (H), ge lao dang (Y)	Herb	Tender stem and leaf	Spices	C - 022	C	Y	0.91
Poaceae	*Dendrocalamus barbatus* var. *internodiradicatus* Hsueh & D. Z. Li	mei huo (D), wo ne (H), lao bie (Y)	Bamboo	Bamboo	Potherb	C - 217	W, C	Y	1
Poaceae	*Dendrocalamus brandisii* (Munro) Kurz	mai wang (D), wo chi (H), lao gan (Y)	Bamboo	Bamboo	Potherb	C - 062	W, C	Y	0.98
Poaceae	*Dendrocalamus giganteus* Munro	mai sang (D), wo pu (H), lao bu bie (Y)	Bamboo	Bamboo	Potherb	C - 176	W, C	Y	0.85
Poaceae	*Dendrocalamus hamiltonii* Nees & Arn. ex Munro	mai bo (D), wo chi (H), lao gang (Y)	Bamboo	Bamboo	Potherb	C - 160	W, C	Y	1
Poaceae	*Dendrocalamus membranaceus* Munro	mai ya (D), cha chu (H), lao gang (Y)	Bamboo	Bamboo	Potherb	C - 181	W, C	Y	0.98
Poaceae	*Dendrocalamus semiscandens* Hsueh & D.Z. Li	mai huo (D), wo nang (H), lao bai (Y)	Bamboo	Bamboo	Potherb	C - 120	W	Y	0.85
Poaceae	*Imperata cylindrica* (Linnaeus) Raeuschel	ya ha (D), yi ke (H), gan (Y)	Herb	Root	Seasonal fruit	C - 032	W	N	0.39
Poaceae	*Indosasa singulispicula* T.H. Wen	mai kong (D), a ka bi (H), lao dong (Y)	Bamboo	Bamboo	Potherb	C - 182	W, C	Y	0.99
Poaceae	*Indosasa sinica* C.D. Chu & C.S. Chao	mai kong (D), a kuo kuo me (H), lao bao (Y)	Bamboo	Bamboo	Potherb	C - 121	W, C	Y	0.96
Poaceae	*Pleioblastus amarus* (Keng) Keng f.	nuo kong (D), a ka bi (H), lao dang ying (Y)	Bamboo	Bamboo	Potherb	C - 207	W, C	Y	1
Poaceae	*Pseudostachyum polymorphum* Munro	nuo hei (D), pe (H), lao di (Y)	Bamboo	Bamboo	Potherb	C - 125	W	N	0.65
Poaceae	*Thysanolaena latifolia* (Roxb. ex Hornem.) Honda	king er (D), me bu (H), ge lao mie (Y)	Herb	Tender stem heart	Potherb	C - 052	W	N	0.83
Polygonaceae	*Fagopyrum dibotrys* (D. Don) Hara	pa ge mong (D), luo zhuo guo (H), gai dui long (Y)	Herb	Tender stem and leaf	Potherb; sour condiment	C - 099	W	Y	0.98
Polygonaceae	*Polygonum chinense* L.	song bie (D), yao me chou ge (H), dang dun (Y)	Herb	Tender stem and leaf	Potherb; sour condiment	C - 061	W	N	0.81
Polygonaceae	*Polygonum viscosum* Buch.-Ham. ex D. Don	han fai (D), e nuo si pi (H), ma liu (Y)	Herb	Tender stem and leaf	Spices	C - 102	C	Y	0.97
Pontederiaceae	*Monochoria korsakowii* Regel & Maack	pa hen (D), an mu (H), gai jiu ding (Y)	Herb	Young leaf	Potherb	C - 185	W	N	0.38
Pontederiaceae	*Monochoria vaginalis* (N. L. Burman) C. Presl ex Kunth	pa hen (D), an mu (H), gai jiu (Y)	Herb	Young leaf	Potherb	C - 133	W	N	0.63
Portulacaceae	*Portulaca oleracea* L.	pa bo liang (D), an ni ze che (H), **ma ci xian** (Y)	Herb	Tender stem and leaf	Potherb	C - 010	W	N	0.37
Primulaceae	*Ardisia solanacea* Roxb.	pa lei(D)	Tree	Tender stem and leaf	Potherb	C - 036	W	N	0.16
Primulaceae	*Embelia ribes* N. L. Burman	an li ge si (H), gang dui biu (Y)	Woody vine	Fruit; tender stem and leaf	Seasonal fruit; salad	C - 044	W	N	0.55
Rosaceae	*Docynia delavayi* (Franch.) Schneid.	guo mian (D), si pi er si (H), biu meng yang (Y)	Tree	Fruit	Salad	C - 107	W, C	Y	0.99
Rosaceae	*Pyrus pashia* Buch.-Ham. ex D. Don	guo kei gai (D), zha shuo si li (H), tang liu guo (Y)	Tree	Fruit; flower	Seasonal fruit; potherb	C - 168	W	Y	0.8
Rubiaceae	*Canthium horridum* Blume	ma kao nei (D), ya dai si (H), ning zhou jian (Y)	Tree	Fruit	Potherb	C - 040	W	N	0.43
Rubiaceae	*Galium elegans* Wallich	guo gan nuai (D)	Herb	Root	Tonic soup	C - 141	W	N	0.03
Rubiaceae	*Paederia foetida* L.	ke dun ma (D), ye kuo bu duo (H), gu fa mei (Y)	Herbaceous vine	Tender stem and leaf	Potherb	C - 126	W	N	0.3
Rutaceae	*Zanthoxylum acanthopodium* DC.	ga (D), mo zi la (H), za bu ga biu (Y)	Tree	Fruit	Spices	C - 142	W	N	0.65
Rutaceae	*Zanthoxylum armatum* DC.	bu ga (D), za la (H), lu ga jian (Y)	Tree	Fruit; tender stem and leaf	Spices	C - 021	W, C	Y	0.96
Rutaceae	*Zanthoxylum myriacanthum* var. *pubescens* (C.C. Huang) C.C. Huang	ma king (D)	Tree	Fruit	Spices	C - 196	W, C	Y	0.19
Rutaceae	*Zanthoxylum scandens* Blume	o ne e bi ne si (H), wei (Y)	Woody vine	Fruit	Spices	C - 108	W	N	0.15
Sabiaceae	*Meliosma arnottiana* (Wight) Walp.	ye jia yang (Y)	Tree	Tender stem and leaf	Potherb	C - 151	W	N	0.28
Saururaceae	*Houttuynia cordata* Thunb.	pa huai (D), ke sa li guo (H), lu lin gai (Y)	Herb	Tender stem and leaf	Salad; tea substitute	C - 047	W, C	Y	0.99
Schisandraceae	*Schisandra henryi* subsp. *yunnanensis* (A. C. Smith) R. M. K. Saunders	ga zi ga nuo (H), dong jing dan (Y)	Woody vine	Fruit	Seasonal fruit	C - 093	W	N	0.24
Scrophulariaceae	*Buddleja officinalis* Maximowicz	luo fan (D), a yi huo si (H), nan wan fan (Y)	Tree	Flower	Dye material to make yellow sticky rice	C - 042	W·	Y	0.86
Solanaceae	*Solanum americanum* Miller	pa ding (D), ku li ba sa (H), gai ge liu (Y)	Herb	Tender stem and leaf	Potherb	C - 083	W	Y	1
Solanaceae	*Solanum nigrum* L.	pa ding (D), ku li ba sa (H), gai ge liu (Y)	Herb	Tender stem and leaf	Potherb	C - 028	W	Y	0.98
Solanaceae	*Solanum spirale* Roxburgh	pa lie (D), ku liao liao ye (H), gai ge lei lu (Y)	Shrub	Tender stem and leaf	Potherb	C - 078	W, C	N	0.36
Solanaceae	*Solanum torvum* Swartz	liang lao (D), mo si kuo (H), ge lan biu (Y)	Shrub	Fruit	Potherb	C - 003	W	Y	0.96
Solanaceae	*Solanum undatum* Lamarck	ke kua (D), ni ga zi (H), ge lan (Y)	Shrub	Fruit	Potherb; salad	C - 139	W, C	Y	0.73
Solanaceae	*Solanum violaceum* Ortega	lian huo (D), si kuo kao yao (H), ge lan di (Y)	Shrub	Fruit	Potherb	C - 163	W	N	0.17
Urticaceae	*Elatostema dissectum* Weddell	dong ma wang (Y)	Herb	Tender stem and leaf	Potherb	C - 119	W	N	0.12
Urticaceae	*Girardinia diversifolia* (Link) Friis	han zhan (D), pa bie (H), mu la (Y)	Herb	Tender stem and leaf	Potherb	C - 143	W	N	0.37
Zingiberaceae	*Alpinia blepharocalyx* K. Schum.	ma ga (D), ge bo bao (Y)	Herb	Inflorescence	Potherb	C - 063	W	N	0.39
Zingiberaceae	*Alpinia galanga* (L.) Willd.	ha (D), mi pi duo pu (H), **jiang miao** (Y)	Herb	Young shoot; rhizome	Potherb; spices	C - 203	W	N	0.72
Zingiberaceae	*Amomum coriandriodorum* S. Q. Tong & Y. M. Xia	guo hao (D), **cao guo** (H), ya ma hao (Y)	Herb	Young leaf	Spices	C - 113	W, C	Y	0.49
Zingiberaceae	*Amomum koenigii* J. F. Gmelin	ma guo (D), mi jie (H), ge bo biu (Y)	Herb	Fruit	Seasonal fruit	C - 186	W	N	0.5
Zingiberaceae	*Amomum maximum* Roxb.	ma guo (D), mi jie (H), ge bo di (Y)	Herb	Fruit	Seasonal fruit	C - 155	W	Y	0.83
Zingiberaceae	*Amomum villosum* Lour.	mang nian (D), **sha ren** (H), **sha ren** (Y)	Herb	Young shoot; fruit	Seasonal fruit; tonic soup	C - 066	W, C	Y	0.55
Zingiberaceae	*Etlingera yunnanensis* (T. L. Wu & S. J. Chen) R. M. Smith	hen dun (D), mi jie (H), ge bo (Y)	Herb	Young shoot	Potherb	C - 216	W	N	0.2
Zingiberaceae	*Zingiber orbiculatum* S. Q. Tong	nuo eng (D), suo ya mi jie (H), ge bo bu (Y)	Herb	Young shoot	Potherb	C - 135	W	Y	0.96

The order of plants in this table is followed alphabetically by family, and then by species

The taxonomic circumscriptions of plant families and species followed the APG IV system

The local names with bold font resembled those in Mandarin or local Chinese dialects

^a^*D* Dai language, *H* Hani language, *Y* Yao language

^b^*WC* wild or cultivated

^c^*MS* market selling, *Y* yes, *N* no

^d^*UV* use value

Among the 211 species, there are 95 herb species (45.0%), 54 trees (25.6%), 38 vines (18.0%), 13 shrubs (6.2%), and 11 bamboos (5.2%) (Table 2). All wild edible plants were also classified by their edible parts (Table 3). The most commonly consumed parts of the plant were tender stem and leaf (91 species, 43.1%), fruit (50 species, 23.7%), flower (29 species, 13.7%), rhizome (15 species, 7.1%), followed by root, bamboo shoot, young leaf, tender stem heart, stem, young shoot, seed, bark, petiole, and inflorescence (Table 3). The rich variety of edible parts collected from different life form species demonstrated that the local communities have gathered a lot of traditional knowledge from their daily consumption of wild edible plants. They could figure out which plant part is safe to eat and get rid of non-edible or poisonous parts. For example, the tuber of *Colocasia* (Araceae) plants, such as *Colocasia esculenta* (L.) Schott and *Colocasia fallax* Schott, is mostly edible as coarse grains, vegetables, or pig feed [43], while the tuber of *Colocasia gigantea* (Blume) Hook. f. is extremely poisonous, not only to insects but also to people. Ingestion of this tuber by accident could cause severe pain in the esophagus and digestive system and the edible part of this plant is thus the petiole but not the tuber [44]. Moreover, many species provide more than two edible parts for the local people, this information could be interpreted as their preference for some species, and could also be useful for the further economic exploration of these wild edible plants.

**Table 2:** Life forms of wild edible plants in Jiangcheng County

Life forms	Records	Percent (%)
Herb	95	45.02
Tree	54	25.59
Herbaceous vine	23	10.90
Woody vine	15	7.11
Shrub	13	6.16
Bamboo	11	5.21

**Table 3:** Edible parts of wild edible plants in Jiangcheng County

Parts used	Records	Percent (%)
Tender stem and leaf	91	43.13
Fruit	50	23.70
Flower	29	13.74
Rhizome	15	7.11
Root	13	6.16
Bamboo shoot	11	5.21
Young leaf	9	4.27
Tender stem heart	9	4.27
Stem	9	4.27
Young shoot	5	2.37
Seed	4	1.90
Bark	2	0.95
Petiole	2	1.90
Inflorescence	2	0.95

### Diversity of usage and preparation methods

As for the usage and preparation methods (Table 4), more than two-thirds of the plants were consumed as potherb (wild vegetables, 67.8%). Potherbs are normally fried or boiled to make a mixed wild vegetable soup. Twenty-four species were used to make salad freshly or after boiling. Besides, 25 wild fruits were consumed as seasonal fruits with 3 of them also used for liquor brewing. The following usages are tonic soup, spice ingredient, sour condiment, liquor brewing, tea substitute, dye material, and nut. During our investigation, we found that local people have some special taste preference for choosing the wild edible plants. For instance, there are 18 species, such as *Litsea cubeba* (Lour.) Pers., *Zanthoxylum armatum* DC., *Zanthoxylum myriacanthum* var. *pubescens* (C.C. Huang) C.C. Huang, *Alpinia galanga* (L.) Willd., and *Amomum coriandriodorum* S. Q. Tong & Y. M. Xia, used as spice ingredients to cook beef or mutton, and 11 species, such as *Spondias pinnata* (L. F.) Kurz, *Begonia augustinei* Hemsl., and *Urceola rosea* (Hooker & Arnott) D. J. Middleton, used as sour condiments to make fish or cook pork soup. Besides the spicy and sour tastes, several species from the Solanaceae and Bignoniaceae families are consumed for their bitter taste. *Acacia pennata* (L.) Willd., having strong bad egg smell, is also used to cook fish soup, mixed wild vegetable soup, or fried egg.

**Table 4:** Usage and preparation methods of wild edible plants in Jiangcheng County

Preparation and use	Records	Percent (%)
Potherb	143	67.77
Salad	24	11.37
Seasonal fruit	25	11.85
Spice ingredient	18	8.53
Tonic soup	21	9.95
Sour condiment	10	4.74
Liquor brewing	9	4.27
Tea substitute	7	3.32
Dye material	3	1.42
Nut	3	1.42

On one hand, wild edible plants provide essential source of food for local people, on the other hand, some of these plants are believed to have health benefits and are utilized as medicine and beverage by the local people in Jiangcheng County. In this study, there are 21 plants used to make tonic soup with chicken or pork. The indigenous villagers used 9 plants to make traditional liquor, which are also frequently consumed to treat stomach or inflammatory disease or to help them to have a healthy and strong body. In Pu’er City, there is a long traditional culture of harvesting and cooking herbal medicines with different meat to make some tonic soup. More than 100 species of medicinal plants were sold on the herb market in Pu’er City during the Dragon Boat Festival (Chinese Duan Wu festival) [45].

The diverse use and preparation methods of wild edible plants in Jiangcheng County indicate that the indigenous people have learned a lot of traditional knowledge about how to adapt well to their natural environment. Their strong connections with nature benefit them, not only by avoiding harmful materials but also providing better taste experiences. These traditional usage and preparation practices raised a wonderfully diversified cooking culture. With the increasing demand for a better and healthy daily life, the consumption of wild edible plants has been increasing and many of them have been collected from the field to serve at local restaurants. The practice of making edible medicinal soup meets the requirements of food nutrition and body health at the same time for the modern world, and attracts more tourists to have a stomach feast in Pu’er City. The traditional knowledge is also valuable for future use of wild edible and medicinal plants in the food industry.

### The use value and traditional knowledge distribution among different generations and ethnic groups

**Fig. 4:**
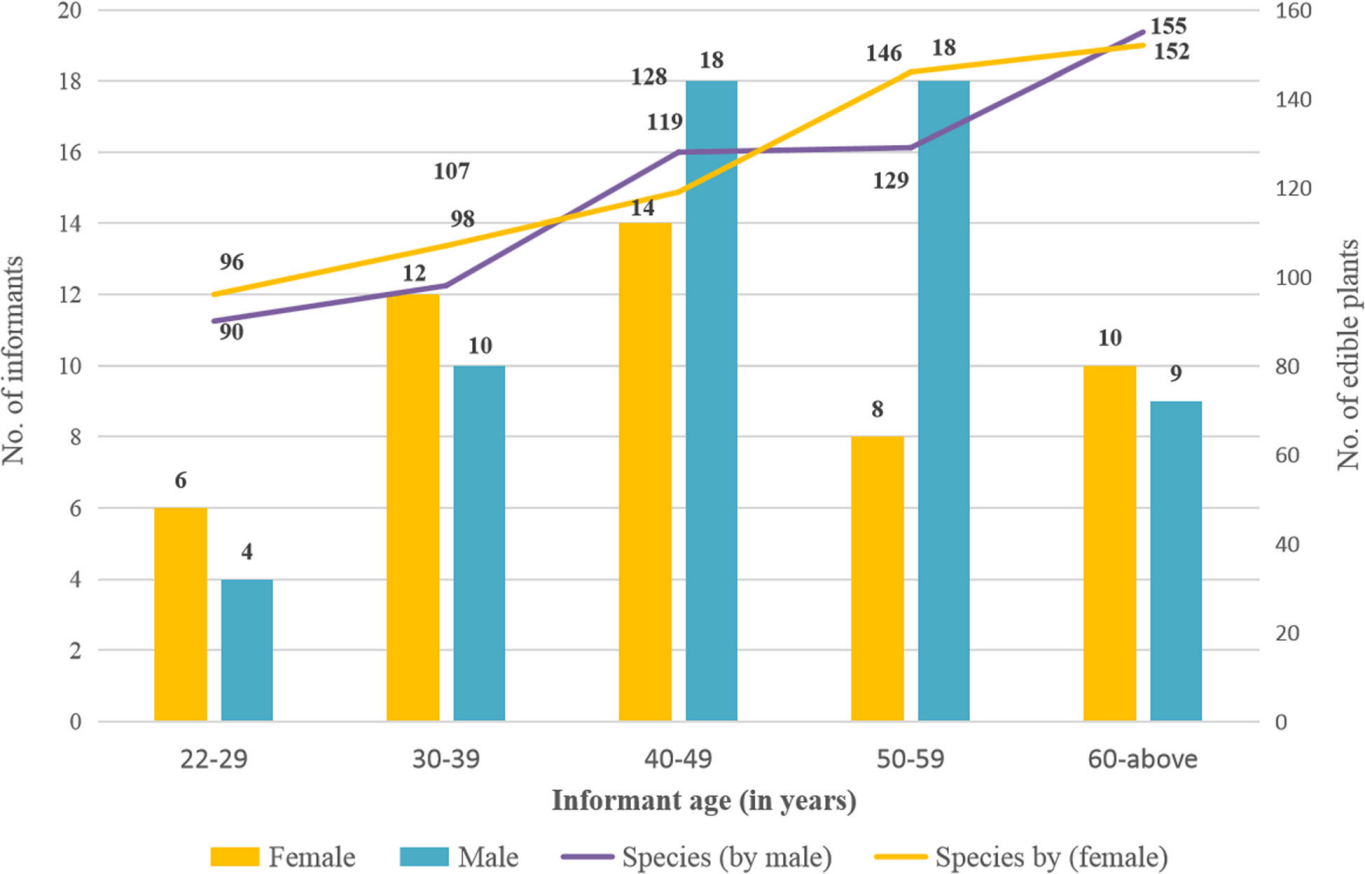
Characteristics of informants and the average number of edible plants consumed by 5 different age groups

**Fig. 5:**
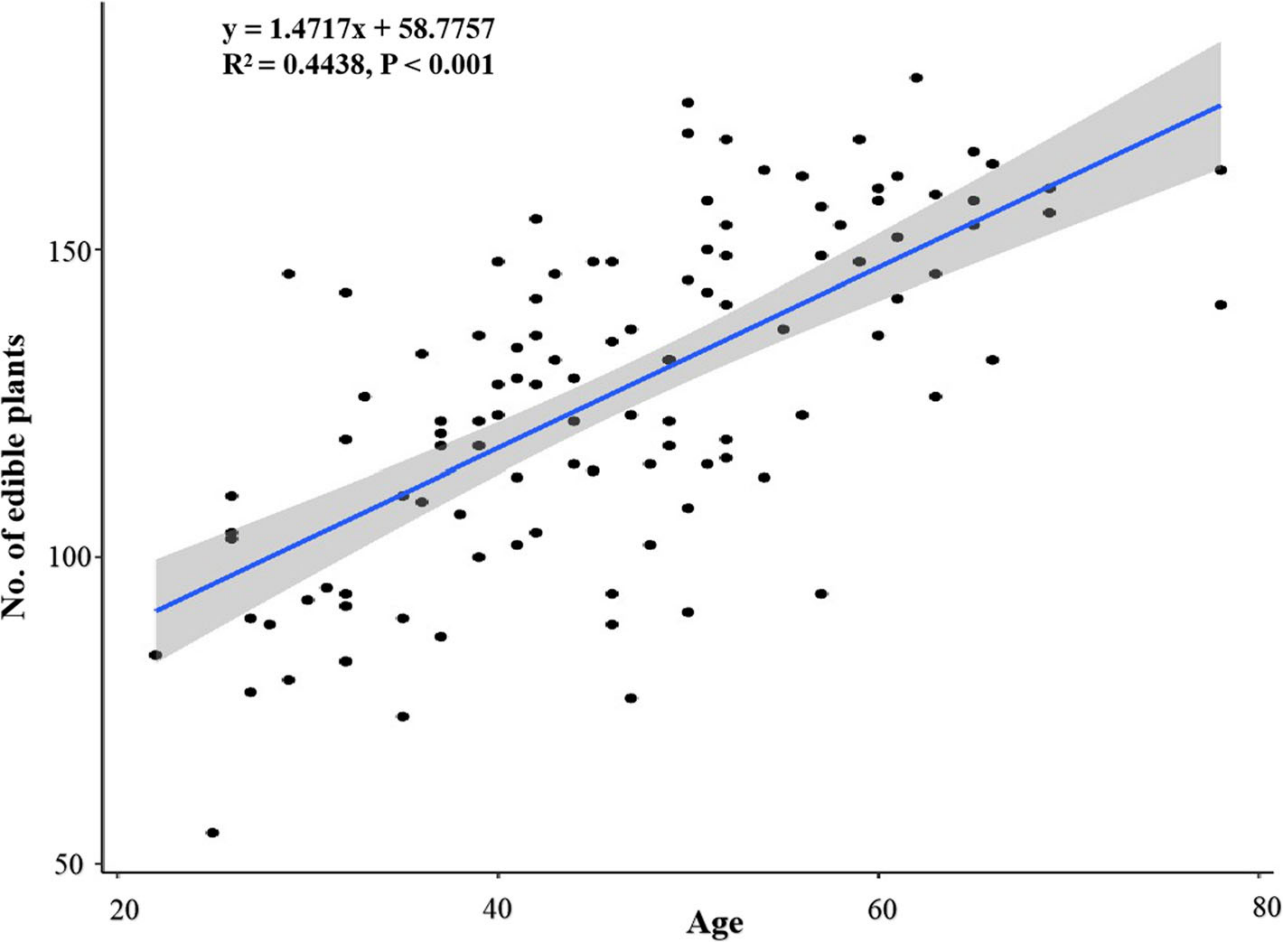
The relationship of informants’ age and the number of mentioned edible plants. In the formula, *y* is the number of recognized edible plant, while *x* is the age of villager

The use values (UV) of each species were calculated to determine their relative importance to local communities (Table 1). Sixty-five species with the highest UV (0.90-1.00) were remarked as the most consumed wild edible plants in Jiangcheng County. The five different age groups of informants (50 males and 59 females) consumed 90 to 155 wild edible species (Fig. 4). Generally, male and female villagers eat similarly for the same age groups and the elder generation owns much more traditional knowledge than the younger generation. Based on the *t* test and uni-variate linear regression, there is significant relationship between respondents’ age and the number of wild edible plants identified (Fig. 5). A previous study showed that age had a positive effect on the number of listed wild food plants [46]. Our study found that, as we expected, the number of wild edible plants increased along with people’s age. The traditional knowledge is under serious threats due to environmental degradation and acculturation, as well as biodiversity loss, and it showed signs of being forgotten and abandoned by the younger generation [15, 35]. This situation is also occurring in Jiangcheng County. The traditional knowledge is handed down to successive generations. With the passing of elderly people, the increased deforestation of natural forest and monoculture of economic plants, and the decreased availability of wild edible plants, the traditional knowledge has high risk of loss among the younger generation in this trans-boundary region. Our study established a baseline for future work on the loss of traditional ecological knowledge among different generations.

**Fig. 6:**
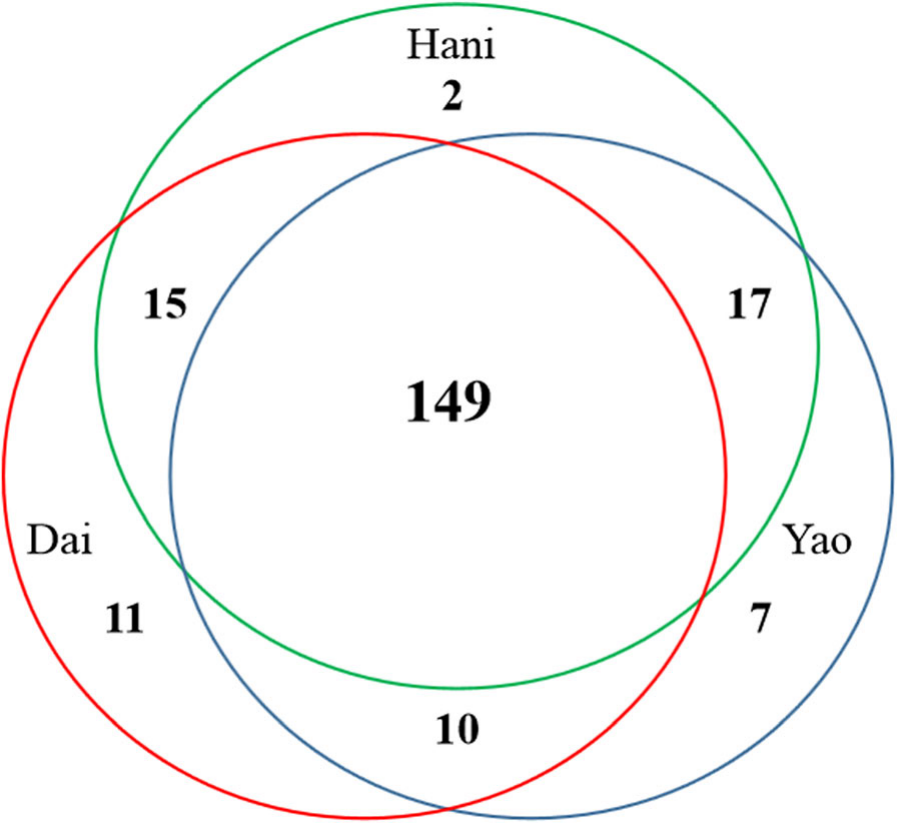
Number of wild edible plants used in three ethnic groups. Red, green, and purple color represent Dai, Hani, and Yao people respectively

Altogether, the local Dai, Hani, and Yao communities in this study consumed a total of 211 wild edible species. The number of wild edible species consumed by each group had a narrow range of 183 to 185 (Fig. 6) and as many as 149 species, accounting for 70.62% of the total wild edible species, were used by all 3 ethnic groups. The comparative analysis by Jaccard index (JI) showed that local Dai, Yao, and Hani communities shared very similar wild edible plants traditional knowledge, with JI values of 89.8%, 92.7%, and 94.9% for Dai and Yao, Dai and Hani, and Yao and Hani respectively. This high similarity might be due to long terms of interactions and communication at similar geographic environment. The high JI values indicate that local people in this trans-boundary region are tightly connected and shared much traditional botanical knowledge with each other. Besides, the ethnic groups also learned some edible plants knowledge from the Han Chinese. For instance, there are 27 species, which are also commonly consumed by the Han Chinese as medicine or vegetable, and these plants’ local names resemble those in Mandarin or local Chinese dialects (Table 1).

Searching around the neighboring areas, there were 284 wild edible species used by Dai, Hani, and Jinuo people in Xishuangbanna Dai Autonomous Prefecture [47] and 224 wild edible plants consumed by Hani people in Honghe Prefecture [35]. When contrasted with these neighboring areas, the wild edible species are quite different, with only 87 overlapping species between Jiangcheng County and Xishuangbanna, and 53 overlapping species for Jiangcheng County and Honghe Prefecture respectively. This result shows that local people could always make a living from the limited circumstances and that the traditional knowledge of wild edible plants is tightly associated with the local environment. The Jaccard index (JI) values were calculated ranging from 13.9 to 21.3%, suggesting that there has high diversity of wild edible plants among these biodiversity hotspot areas.

### Cultivation, market, and conservation status in the studied trans-boundary region

Besides their abundant experiences about harvesting wild edible plants from forest, local Dai, Hani, and Yao people also have a very rich traditional knowledge of introduction and cultivation of wild plants. There are 68 plants cultivated by the local people in Jiangcheng County (Table 1). In the neighboring Xishuangbannan, Dai people have cultivated 315 plants in their villages, with 69 species used as medicines, fruits, vegetables, spices, horticultural flowers, and construction woods [48]. Seventy-nine kinds of folk utilizable plants of swidden agroecosystem that belong to 38 families and 64 genera were cultivated by Hani people, and used as firewood, food, fruit, fodder, beverage, condiment, and textile [49].

Wild edible plants not only supply the daily materials needed but also play an important role in ethnic groups’ cash income. There are 117 wild edible plants sold at the local markets by the local people in Jiangcheng County (Table 1), and 146 wild vegetable species were found to be sold at the local markets and restaurants in Xishuangbanna [47]. On one hand, the huge market demands for wild edible plants contribute to thickening the wallet for the local people. On the other hand, it has been stimulating the increase of pressure on wild collection from the wild, with potential implications for biodiversity conservation. For instance, *Panax zingiberensis* C.Y. Wu & K.M. Feng, which was cooked by the local people with chicken to produce healthy soup, and mixed with traditional wine to make tonic liquor, has already been listed as endangered species, and may need more conservation efforts to resolve this potential over-harvesting pressure.

Habitat loss is another main threat to conserve the endangered or rare wild edible plants. During our investigation, the shift cultivation land and nearby forests have almost completely been transformed into rubber plantation. We found the rice fields were rented by the businessman to grow banana, chili, watermelon, kidney bean, and some other cash crops almost during the whole year. The original rice fields, which are normally full of water and an important wetland for many creatures, have now become relatively dried farm lands. The decrease areas of rice paddy caused severe loss of wetland habits, biodiversity, and cultural diversity in Jiangcheng County. For example, *Brasenia schreberi* J.F. Gmel. could be very easily found at the rice field and other wetland area and had some semi-cultivation practice [47], while it almost disappeared because of the loss of rice fields and change of land use for cash crops. Moreover, among the interviewed 109 villagers, only 27 respondents knew still how to eat this species. These were all elder people with an average age of 57.5 years. This result suggests the younger generation have already lost the traditional knowledge about this edible species, due to the rapid decrease and shrink of wetland habitats. Thus, we highlight that more conservation concern and efforts should also be paid to the tropical wetland areas.

Although, the decrease of wild edible plants still happens because of environmental change and human negative effects, there are also case of edible species increases due to human cultural exchange. *Dendrobium* species are usually consumed as medicine and crushed freshly to treat scald disease by local Dai people [50]. While, *Dendrobium* species are famous and expensive traditional tonic medicine to rescue lives by Han people in the middle and east part of China. In the 1990s, there was a huge increase in demand of wild *Dendrobium* plants, and the related cultivation industry boomed in Southwest China for its suitable climate and lower investments. Local people learned that *Dendrobium* species not only have external and medicinal use but also could be cooked and eaten for health benefits during their communication with the outside businessman. There were 107 orchid species sold at Xishuangbanna market with *Dendrobium* plants as the main traded species [51]. The culture exchange enriched the dish list of the local people, while it also contributed the increase harvesting and conservation pressure of endangered *Dendrobium* species because of the preference of wild products by the locals. Our results thus indicate that culture exchange could increase the culture diversity but might have more conservation pressure on endangered species, and ethnobotanical data about the use frequency and consumption demands of the endangered edible species should be included and considered when we evaluate the conservation status of the threatened species.

### Important role of traditional knowledge for local communities and forest ecosystem services

The forest plays an important role for local communities. Local Dai people have a well-known proverb saying that only where there is fine forest, there is water, farming land, food, and people can thrive. This classic ecological belief ranked the forest in an extremely high position, and made a positive contribution to the biodiversity conservation in this region [52, 53]. Based on the guidelines from specifications for assessment of forest ecosystem services in China [54], the estimated total value of forest ecosystem services in Pu’er City was 247,785 billion yuan per year, with the per unit area value of forest ecological service at 85,500 yuan per hectare per year [55]. These assessments were necessarily very simplified, usually focusing on a few, easily quantified services, and failed to include the services which are of most importance to local people and could therefore lead to incorrect policy decisions [56]. Jiangcheng County’s forest cover increased rapidly from 43 to 68% during 1997 to 2018, according to the public data from the local government [7]. The availability of wild edible plants obtained from the forest, however, seems to have decreased according to our result and the description by the elder informants. Globally, the Aichi Biodiversity Target 11 (to protect at least 17 percent of terrestrial area by 2020) has been exceeded for forest ecosystems, but deforestation and forest degradation continue to take place at alarming rates and contribute significantly to the ongoing loss of biodiversity [57].

Therefore, on one hand, we suggested that we should equally evaluate the quantity and quality of the forest cover rate, and pay more attention to the negative effects of mono-culture forest plantations, such as rubber, on the traditional knowledge conservation and inheritance. On the other hand, ethnobotanical data on the value of the forest for providing the wild edible plants and other non-timber forest products to the local communities as well as the feedback effect from traditional knowledge and cultural diversity to forest conservation should be added into the specifications for assessment of forest ecosystem services.

### Traditional cultivated plant genetic resources and diversified agriculture

Local ethnic groups have a long tradition of introducing their preferred wild plants into farm lands and homegardens. The cultivated wild species are very important plant genetic resources (PGR) for the development of cash crops. Local Dai communities, cultivated 204 species for edible, medicinal, ornamental, and religious purposes, have a very close relationship with the formation and development of PGRs and play an important role in the conservation and utilization of PGRs [58].The UN’s intergovernmental panel on climate change (IPCC) in Geneva issued a special report on climate change and land, pointing out that human activities and climate change will place land resources under huge pressure and that sustainable land and forest management, could prevent and reduce land degradation, maintain land productivity, mitigate the adverse effects of climate change to some extent, and conserve the precious land and ecosystems at the same time [59]. A recent discovery revealed that altering the cropping pattern from intensive monoculture to diversified agriculture, could help to withstand the climate change, protect vital wildlife, and alleviate the long-term loss of biodiversity outside natural protected areas in the future [60]. Besides, increasing plant species diversity could promote beneficial trophic interactions between insects and plants, ultimately contributing to increased ecosystem services [61].

Thus, we suggested that more wild edible plants could be introduced and cultivated in the nearby protected areas, farming land, rubber forest, tea plantations and village owned forest, homegardens, and any suitable sites to build corridors or ex situ reserve areas for some important, rare, medicinal, and edible plants, conserve more plant genetic resources and establish a diversified agriculture. Moreover, modern plantation technology should also be updated, and the cultivation and domestication of some preferred wild edible plants should be strengthened by cooperating with some food industries to reduce field collection, increase economic income and contribute to the sustainable development of local communities.

### Top 30 wild edible plants for better conservation, understanding, and sustainable utilization in China, Laos, and Vietnam trans-boundary region

**Fig. 7:**
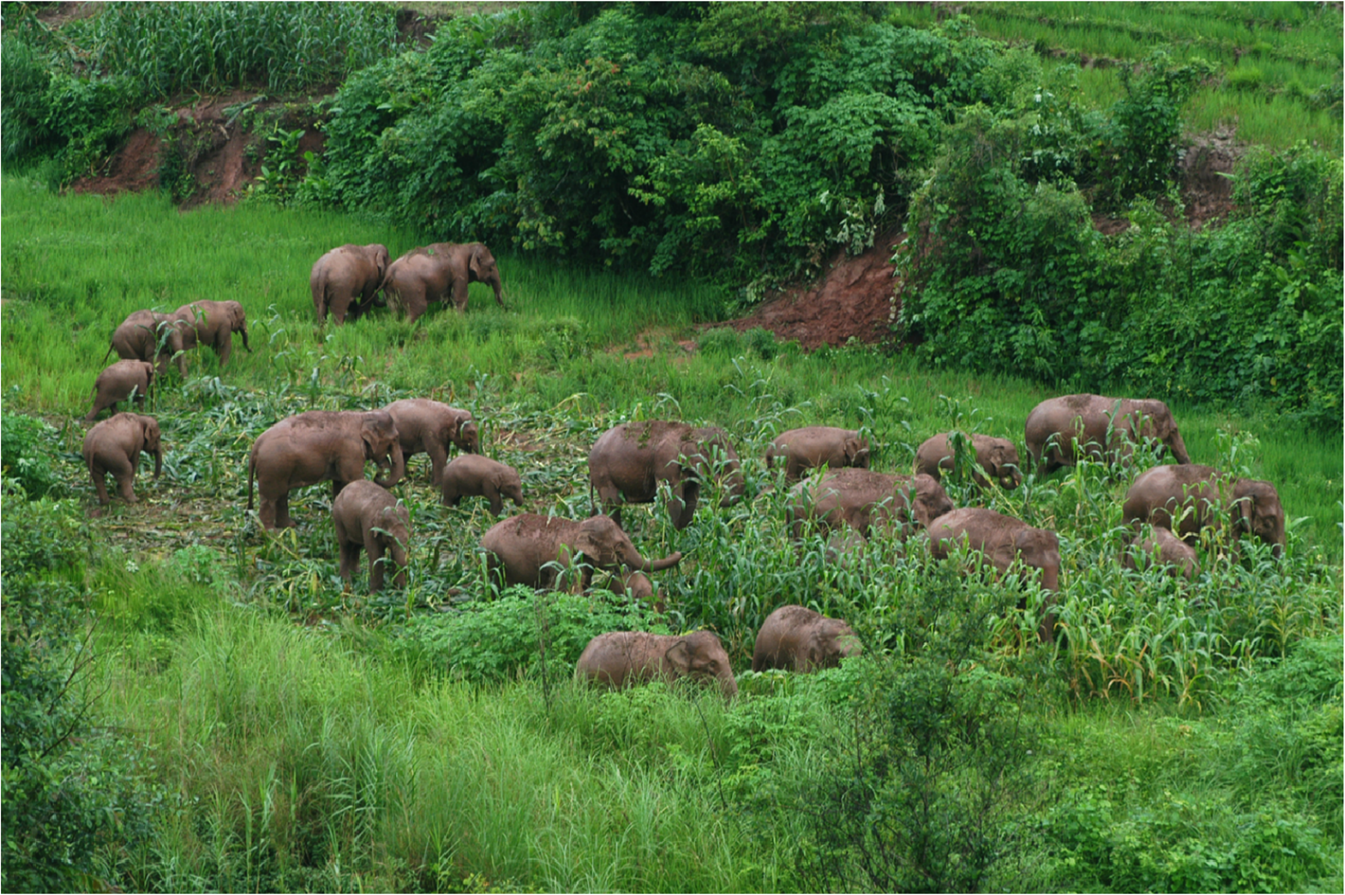
A group of Wild Asian elephants feasting the corn and rice cultivated by local villagers nearby Jiangcheng County, Southwest China. Photographed by Mr. Shishun Zhou

Besides the threats from climate change, plantation and livelihood transformation, over-harvesting and alarming loss of traditional knowledge, the local people also have to face human-elephant conflict for there have around 44 wild Asian elephant (*Elephas maximus*) individuals in Jiangcheng County (Fig. 7). With the potential increasing of elephant’s population and expansion of its distribution, human-elephant conflict would be more serious due to continuous insufficient food, habitat loss, and fragmentation [32, 62, 63]. The wild plants are important and reliable food sources both for human and elephant. Among the reported 240 forage plants for wild Asian elephant in Southwest China [64], there are at least 44 overlapping wild edible plants both for human and elephant in Jiangcheng County. Thus, local ethnic people might have higher accident risk with wild Asian elephant when they are both trying to harvest the same or similar wild edible plants at the same time. Nevertheless, establishing a food source base with fast-growing, and high biomass indigenous plants has proven to be one of the effective ways to solve this problem [65]. However, how to choose more suitable plants to introduce and cultivate in the elephant food source base still lacks practice and research data.

With limited land and investments, it is difficult to overcome all these mentioned problems, but we could use a multidimensional way to solve or minimize these issues. Based on our ethnobotanical survey, the data from threatened species list of China’s Higher Plants and the IUCN Red List, the published food plant list for Asian Elephant, the Subject Database of China Plant and the calculated UV score, the top 30 most important wild edible plants (Table 5) were identified and recommended to be further cultivated and expanded in some local villages. These highlighted plants include 15 threatened or endangered species, 17 species with UV value over 0.9 and 19 species consumed by both humans and elephants. The environmental, cultural, and religious benefits of the forest are generally recognized by the local people [66]. By learning the ethnobotanical knowledge from the ethnic groups and encouraging them to cultivate more plants, especially the endangered species, in the community land and individual households retained forest, as well as their homegardens could open a new channel for connecting the fragmented forest as a whole, then contributing to conservation and the sustainable use of natural resources.

**Table 5:** The top 30 most important wild edible plants recommended for cultivation, conservation, and sustainable use

Family name	Scientific name	TSLCHP^a^	NPLC^b^	IUCN^c^	Elephant^d^	UV^e^
Anacardiaceae	*Mangifera siamensis* Warbg. ex Craib	EN				1.00
Anacardiaceae	*Mangifera sylvatica* Roxb.	EN	2	EN	Y	0.94
Anacardiaceae	*Spondias pinnata* (L. F.) Kurz				Y	0.94
Apocynaceae	*Amalocalyx microlobus* Pierre				Y	0.98
Araliaceae	*Panax zingiberensis* C.Y. Wu & K.M. Feng	EN	1	EN		0.14
Arecaceae	*Caryota obtusa* Griffith	VU	2		Y	0.49
Bignoniaceae	*Mayodendron igneum* (Kurz) Kurz				Y	0.99
Bignoniaceae	*Oroxylum indicum* (L.) Bentham ex Kurz				Y	0.97
Cabombaceae	*Brasenia schreberi* J.F. Gmel.	CR	1	CR		0.25
Cycadaceae	*Cycas pectinata* Buchanan-Hamilton	VU	1	VU	Y	0.12
Dilleniaceae	*Dillenia indica* L.	EN			Y	0.83
Elaeocarpaceae	*Elaeocarpus austroyunnanensis* Hu	VU				0.45
Euphorbiaceae	*Baccaurea ramiflora* Loureiro				Y	0.99
Fabaceae	*Acacia pennata* (L.) Willd.				Y	0.99
Fabaceae	*Bauhinia variegata* var. *candida* (Roxb.) Voigt				Y	0.99
Menispermaceae	*Parabaena sagittata* Miers				Y	1.00
Moraceae	*Ficus auriculata* Lour.				Y	0.93
Moraceae	*Ficus virens* Aiton				Y	0.93
Moraceae	*Morus alba* L.				Y	0.93
Orchidaceae	*Dendrobium chrysanthum* Wall. ex Lindl.	VU	1	VU		0.52
Orchidaceae	*Dendrobium crepidatum* Lindl. ex Paxton	EN	1	EN		0.51
Orchidaceae	*Dendrobium cucullatum* R. Br. ex Lindl.	VU				0.52
Orchidaceae	*Dendrobium devonianum* Paxton	EN	1	EN		0.51
Poaceae	*Dendrocalamus hamiltonii* Nees & Arn. ex Munro				Y	1.00
Poaceae	*Pleioblastus amarus* (Keng) Keng f.				Y	1.00
Polygonaceae	*Fagopyrum dibotrys* (D. Don) Hara				Y	0.98
Rutaceae	*Zanthoxylum myriacanthum* var. *pubescens* (C.C. Huang) C.C. Huang	VU				0.19
Solanaceae	*Solanum torvum* Swartz				Y	0.96
Lamiaceae	*Gmelina arborea* Roxb.	VU				0.36
Zingiberaceae	*Etlingera yunnanensis* (T. L. Wu & S. J. Chen) R. M. Smith	VU	2			0.20

^a^*TSLCHP* Threated Species List of China’s Higher Plants, *EN* endangered, *VU* vulnerable, *CR* is critically endangered, “-” means not included or data deficiency

^b^*NPLC* National Protection level in China

^c^*IUCN* is the IUCN Red List of Threatened Species

^d^Asian elephant forage plants; *Y* yes

^e^*UV* use value

Furthermore, from better protection of wild edible plants view, there is urgent need for policymakers to enhance the government coordination in this trans-boundary region [67, 68], and reinforce the monitoring and management of rare or endangered plants traded in local markets, to popularize the biodiversity conservation laws and to promote the awareness of the value of traditional knowledge. From the sustainable utilization and development of wild edible plants view, local governments could continue to make their three times per month’s traditional market day (every 1st, 11th, 21st of each month) more famous of typical ethnic culture characters by encouraging local ethnic groups to sell more cultivated wild plants there. The trans-boundary good trade fair would be another platform for local communities to demonstrate their unique culture and to increase the nationalities’ self-identification, then contribute to the conservation and inheritance of traditional knowledge for the trans-boundary ethnic groups.

## Conclusion

An ethnobotanical study on wild edible plants used by three trans-boundary ethnic groups was conducted in Jiangcheng County, Pu’er City, Southwest China. A total of 211 wild edible plants and their traditional knowledge were documented in this study. Our results show that three trans-boundary Dai, Hani, and Yao people have plentiful traditional knowledge on the utilization of wild edible plants with diversified eating parts, preparation methods, and use purposes. Local people not only collect the edible plants from wild, but also cultivated and sold them in the markets. However, many of these wild edible plants were only frequently mentioned by the elder informants and there is an alarming risk of losing the traditional knowledge among younger generations. Endangered plants distributed at the wetland or sold at the market, such as *Brasenia schreberi* J.F. Gmel., *Panax zingiberensis* C.Y. Wu & K.M. Feng, and *Dedrobium* species, deserve more conservation efforts. Based on our results, the top 30 most important wild edible plants were highlighted to be further cultivated and expanded in some local villages.

In conclusion, wild edible plants play an important role in local people’s daily life, and the ethnobotanical information of the wild edible plants collected from ethnic groups could provide key scientific data to promote the traditional cultural value among the young generation and relief the stress of human-environment conflict. By referring to the traditional knowledge from the ethnic groups and encouraging them to make a diversified cultivation of wild edible plants in the community land and individual households, as well as their homegardens could launch a new bridge for wild plants to be more profitable cash crops, contribute to the sustainable use of natural resources, and conserve the endangered species in this trans-boundary region.

## Data Availability

All data generated or analyzed during this study are included in this published article.
